# Progress in the Optical Sensing of Cardiac Biomarkers

**DOI:** 10.3390/bios13060632

**Published:** 2023-06-07

**Authors:** Cristina Polonschii, Monica Potara, Madalina Iancu, Sorin David, Roberta Maria Banciu, Alina Vasilescu, Simion Astilean

**Affiliations:** 1 International Centre of Biodynamics, Intrarea Portocalelor 1B, 060101 Bucharest, Romania; cpolonschii@biodyn.ro (C.P.); sdavid@biodyn.ro (S.D.); rbanciu@biodyn.ro (R.M.B.); 2 Nanobiophotonics and Laser Microspectroscopy Center, Interdisciplinary Research Institute in Bio-Nano-Sciences, Babes-Bolyai University, T. Laurian Str. 42, 400271 Cluj-Napoca, Romania; monica.potara@ubbcluj.ro (M.P.); simion.astilean@ubbcluj.ro (S.A.); 3 “Professor Dr. Agrippa Ionescu” Clinical Emergency Hospital, 7 Architect Ion Mincu Street, 011356 Bucharest, Romania; madalina.iancu@gmail.com; 4 Faculty of Chemistry, University of Bucharest, 4-12 “Regina Elisabeta” Blvd., 030018 Bucharest, Romania

**Keywords:** cardiovascular diseases, optical assay, biosensors

## Abstract

Biomarkers play key roles in the diagnosis, risk assessment, treatment and supervision of cardiovascular diseases (CVD). Optical biosensors and assays are valuable analytical tools answering the need for fast and reliable measurements of biomarker levels. This review presents a survey of recent literature with a focus on the past 5 years. The data indicate continuing trends towards multiplexed, simpler, cheaper, faster and innovative sensing while newer tendencies concern minimizing the sample volume or using alternative sampling matrices such as saliva for less invasive assays. Utilizing the enzyme-mimicking activity of nanomaterials gained ground in comparison to their more traditional roles as signaling probes, immobilization supports for biomolecules and for signal amplification. The growing use of aptamers as replacements for antibodies prompted emerging applications of DNA amplification and editing techniques. Optical biosensors and assays were tested with larger sets of clinical samples and compared with the current standard methods. The ambitious goals on the horizon for CVD testing include the discovery and determination of relevant biomarkers with the help of artificial intelligence, more stable specific recognition elements for biomarkers and fast, cheap readers and disposable tests to facilitate rapid testing at home. As the field is progressing at an impressive pace, the opportunities for biosensors in the optical sensing of CVD biomarkers remain significant.

## 1. Introduction

Cardiovascular diseases (CVD) represent the leading cause of mortality and morbidity worldwide [1], being responsible for approximately 17.9 million deaths each year [2]. Moreover, during the last 30 years, the World Health Organization reported a gradual increase in the number of patients with CVD, not only in developed but also in developing countries [3]. Unfortunately, not only older people are affected by CVD, especially nowadays, when sedentarism, obesity, smoking, diabetes, stress, hypertension and many other risk factors are at high levels. Despite all the progress in the field of cardiology, mortality and morbidity in relation to cardiovascular diseases are still very high. Continuous efforts are being made by professional organizations, authorities and research institutes to improve primary prevention and establish simple algorithms in order to facilitate the early and accurate diagnosis of cardiovascular diseases.

The most frequent and life-threatening cardiac pathologies are acute and chronic coronary syndromes and heart failure, accounting for more than 75% of all cardiac emergency room presentations and hospitalizations. “Classical” or newer investigations are used to obtain rapid and precise diagnosis and staging in every case.

After medical history and clinical examination, most cases presenting dyspnea, fatigue, leg swallowing and/or chest pain are usually administered an electrocardiogram (ECG), blood tests, a chest radiography and an echocardiography as soon as possible. Depending of these results, several patients need further, more or less invasive tests such as: an ECG or echocardiography stress test, coronary calcium score quantification by computer tomography (CT), coronary CT angiography, 24 h ECG monitoring, chest CT, cardiac magnetic resonance, coronary angiography or cardiac catheterization. These are the main tests used by cardiologists to establish an accurate diagnosis and an adequate therapeutic approach [4,5]. Cardiac biomarkers of CVD play key roles in diagnosis, risk assessment, treatment, and supervision. The blood levels of cardiac troponin I (cTnI) and blood natriuretic peptides (BNP or NT-proBNP) are the most useful indicators for diagnosing both ischemic heart disease and heart failure. They are equally useful for evolution monitoring and treatment adjustment. Specific analytical tests are available in every emergency room and cardiology department. However, they are far from being perfect due to a variable percentage of false-positive or false-negative tests and sometimes due to the duration of obtaining results in relation to the critical urgency in some cases.

The methods currently used in clinical laboratories exploit the selectivity and strong binding affinity between an antigen (i.e., the biomarker) and an antibody. The specific recognition event is translated into a chemiluminescence, fluorescence, colorimetric or radiometric signal. Various robust, ultrasensitive methods and benchtop equipment are available in clinical laboratories and are complemented by simpler, point-of-care tests and devices available commercially and used in hospitals and care centers [6]. Nonetheless, there is room for further improvement in the specificity, sensibility or speed of methods for cardiac biomarkers and for less invasive determinations to achieve very early diagnosis and the best treatment for all patients.

Along with standard, laboratory-based methods for the biochemical analysis of a panel of cardiac biomarkers, various point-of-care devices, including those based on biosensors, have been proposed for the diagnostic and monitoring of cardiovascular diseases (CVD).

Biosensors are promoted as a solution for rapid, specific and cost-effective analysis, amenable for on-site or point-of-care (POC) analysis and addressing various complex biological matrices, including, e.g., saliva for non-invasive testing. As per 2020 data [7], the analytical performances of POC devices approach those of standard methods used in centralized laboratory settings; however, documented proof of performance for whole blood analysis and randomized clinical trials are still needed to validate these devices as real alternatives to the currently used methods.

The present work reports on the advances in optical biosensors for CVD developed in the past 5 years, placing emphasis on selected CVD biomarkers. The main aim is to highlight several original approaches that may guide or inspire a researcher pursuing the development of new detection methods for CVD biomarkers. In addition, the review discusses the remaining challenges and perspectives in this field.

The focus on optical detection methods is justified by the competitive advantage in comparison with other detection modes with equally great sensitivity (e.g., electrochemical [8,9]). Specifically, optical devices are compatible with either existing equipment or low-cost solutions, e.g., enabling their integration with smartphone detection or with the widely used lateral flow devices.

In optical biosensors, the biological recognition event wherein a CVD biomarker is bound to a specific antibody, aptamer, peptide, cell, etc., is detected by changes in the input light, which manifests as changes in the amplitude, frequency, phase or polarization. There are five categories of optical biosensors based on photoluminescence (fluorescence in particular), chemiluminescence, colorimetry, spectroscopy methods (surface enhanced Raman spectroscopy and infrared spectroscopy) and surface plasmon resonance (SPR) as detection modes. Electrochemiluminescence, where the optical readout relies on luminescence generated via an electrochemical process, combines specific features of electrochemical and optical methods and will be discussed as well.

Various biomarkers have been proposed in relation to CVD diagnosis and monitoring [10]. A pragmatic overview of biosensors for CVD, from a medical professionals’ perspective, lists cardiac Troponin I (cTnI), brain natriuretic peptide (BNP), its precursor N-terminal proBNP (NT-proBNP) and D-dimer as the most useful CVD indicators [6]. Undoubtedly, there are many other compounds that, even if less specific by themselves, help establish the risk or next steps in the treatment of a CVD patient. Multiplexed detection of cardiac biomarkers, a trend in recent years [9], has higher accuracy for CVD diagnostics than single tests alone. Consequently, several additional biomarkers will be briefly discussed in this work, in particular, myoglobin, creatinine kinase myocardial band (CK-MB, an isoform of creatinine kinase) and C-reactive protein (CRP).

The effervescence in the field of POC devices is manifested in various directions of development, from monitoring heart failure patients at home (e.g., a lateral flow immunoassay (LFIA) for NT-proBNP coupled with a portable reader and with IoT implementation [11]) to non-invasive monitoring tools to be used in emergency service vehicles or emergency (e.g., a transdermal device for the detection of cardiac troponin by attenuated total reflection Fourier transformed infrared spectroscopy (ATR-FTIR) [12]). In addition, various commercially available POC devices and sensitive standard methods and pieces of equipment are routinely used in clinical laboratories and emergency units in hospitals [13] (Table 1). These tests are also used as references for the validation of newly proposed bioanalytical devices for CVD detection.

Where do optical biosensors fit in this landscape? To answer this question, in the following section, the main features and analytical performances of optical biosensors developed in the past 5 years are discussed further in relation to the detection method.

## 2. CVD Biomarkers

The first biomarker released after the damage occurs to myocardial muscle cells is myoglobin. Myoglobin was indicated to be a potential biomarker for AMI, the maximum quantity after myocardial cell death being produced within 4 to 6 h from the infarction event [21]. In this interval, the myoglobin levels in blood rise to 70–200 ng/mL, while normal values are reported to be 6–85 ng/mL [22]. If the patient’s blood is analyzed after this period of time, the concentration of myoglobin is no longer relevant as it reverts to its base value. Moreover, the myoglobin concentration is not a very specific CVD indicator; it can rise when other medical conditions occur, such as inflammation, renal failure or skeletal muscular dystrophy [23].

B-type natriuretic peptide (BNP), cardiac troponin I (cTnI), and C-reactive protein (CRP) are released after myoglobin and are specific markers for coronary events. BNP is useful for the diagnosis of heart failure (HF) and for the prognosis in patients with acute coronary syndrome (ACS). CRP is an important prognostic indicator of cardiovascular risk and ACS. Cardiac troponin I (cTnI) has become a standard marker for the detection of acute myocardial infarction (AMI). During the heart infarction, the cardiac troponin T (cTnT) is immediately released into the bloodstream. The N-terminal portion of the B-type natriuretic peptide (NT-proBNP) is a sensitive marker for assessing the risk of heart failure risk. Elevated concentrations of these cardiac markers in serum are associated with recurrent CVD events and higher death rates [24].

Historically, cardiovascular diseases were primarily diagnosed from ECG using graphical abnormalities and elevated quantities of cardiac muscle enzymes, namely CK-MB. Creatinine kinases are enzymes that catalyze the transfer of a phosphate group from creatinine phosphate to adenosine diphosphate, thus leading to the formation of adenosine triphosphate. The enzyme is the combination of two subunits named “M” and “B”, which give the complete name “CK-MB”. The normal percentage of total creatinine kinase is 3–5%, while the peak level ranges from 15 to 30% within 3 to 8 h from the myocardial cell wall injury event [25].

After it was established that some AMI-suffering patients did not have increased serum levels of CK-MB, it was essential to find another biological marker. In muscle fibers, actin and myosin filaments interact to trigger muscle contraction. Because this process is mediated by the troponin–tropomyosin complex, troponin proteins were taken into consideration as cardiac biomarkers. More specifically, serum troponin I (cTnI) was shown to be efficient in the diagnosis of cardiac damage [26,27].

CVDs can have an inflammation component and several biomarkers were proposed to be relevant in this respect, for instance, tumor necrosis factor alpha (TBFα), interleukin-6 (IL-6) and C-reactive protein (CRP) [28].

Elevated basal levels of CRP in blood serum might indicate hypertension and a high risk of CVD. The normal concentration of CRP is 10 mg/L, but when severe inflammation exists in the body, the value can be about 200 mg/L [29]. CRP is released after myoglobin and returns to normal blood levels after 19 h following an acute inflammation event [29].

BNP has several biological effects, including decreased vascular resistance, dilation of blood vessels, and decreased blood volume and pressure through increasing urine production. These effects are linked to BNP’s release upon myocardial stretching [30]. BNP is produced from the enzymatic cleavage of the proBNP precursor molecule, together with NT-proBNP, which is physiologically inactive [31]. The normal level of BNP in blood is around 20 pg/mL and increases in pathological events up to 0.1 ng/mL. The lifetime of BNP in blood is only 20 min. The quantity of NT-proBNP in the bloodstream is 5–10 times higher than the amount of BNP [32]. BNP and NT-proBNP are both considered excellent indicators of heart failure.

IL-6 is an inflammatory biomarker that is involved in regulating cell processes such as proliferation, differentiation and maturation. Congestive heart failure and left ventricular dysfunction are CVDs that are associated with increased concentrations of IL-6 [33]. The levels of IL-6 in blood evolve from less than 0.7 pg/mL in healthy patients to up to 15 ng/mL in heart failure and can even reach 50 ng/mL in case of severe inflammation [33,34,35].

TNFα is a cytokine, an intracellular chemical messenger involved in inflammatory processes. Even if this biomarker is not specific to CVDs, it predicts mortality in patients with heart failure. TNFα’s normal range in serum is between 0.4 and 1 pg/mL, while levels of 1–10 pg/mL indicate heart failure and myocardial infarction [34,35].

## 3. Optical Biosensors for CVD: Recent Examples

### 3.1. SPR-Based Biosensors

Surface plasmon resonance (SPR) is an optical measurement method that utilizes the phenomenon occurring in a thin metallic layer (e.g., gold) positioned at the interface of two media with different refractive indices, illuminated by a light beam at a particular angle of incidence. Surface plasmon resonance (SPR) is an optical measurement method that utilizes the phenomenon occurring in a thin metallic layer (e.g., gold) positioned at the interface of two media with different refractive indices, illuminated by a light beam at a particular angle of incidence.

At a particular angle of the incident light (called the “resonance angle”), the excited collective electrons in the surface layer resonate and adsorb part of the light. This is seen as a minimum (“dip”) in the reflectivity/wavelength curves, i.e., the representation of reflectivity/wavelength versus the incidence angle. SPR is very sensitive to minute changes in the refractive index of the media above the metallic layer, which can be monitored in real time and in a label-free manner. These changes are seen as a modification in the angle, phase or amplitude of the reflected light and are related to interactions that occur in close vicinity of the metallic layer such as antibody–antigen or aptamer–analyte reactions [36]. Usually, the sensor surface is immobilized with a layer of specific antibody or aptamer ligand and the target molecule is injected over the surface. As the target molecules bind to the ligands, the refractive index changes proportionally to the accumulated mass or modifications in the structure of the active layer [37] and is detected and quantified by SPR. Most of the current SPR detection schemes are based on angular or wavelength interrogation (by monitoring the shift of SPR dips), and on intensity interrogation (by monitoring the intensity under a fixed angle of incidence or wavelength). They have a typical resolution of 10^−5^–10^−7^ refractive index units (RIU) [38], with the angle resolved-SPR having an intrinsic higher sensitivity than single-angle SPR [39]. However, angle-resolved SPR devices generally require expensive equipment, complicated optics, and precise alignment of the components, features that hinder the development of a portable device.

Surface plasmon resonance (SPR)-based assays were successfully used for the detection of numerous biomarkers in plasma and serum [40], including CVD biomarkers (Table 2).

An SPR biosensor immobilized with anti-cardiac troponin I monoclonal antibody was developed by [41] and used for the detection of cTnI in aqueous solution and patient serum and kinetic analysis. The gold surface of the SPR chip was functionalized with polyacrylic acid and polydiallyldimethylammonium chloride. Using amino-coupling, the anti-cardiac troponin I monoclonal antibody was immobilized and then the SPR biosensor was blocked with BSA. The limit of detection and limit of quantification were calculated as 0.00012 ng/mL and 0.00041 ng/mL, respectively. The SPR measurements were performed using the commercial SPR imaging system GenOptics, SPRiLab (Orsay, France).

A strategy for cTnI detection was developed by constructing a universal biosensing interface composed of zwitterionic peptides and aptamers [42]. The peptides were self-assembled onto gold chips, some of them being biotinylated. The cTnI-specific biotinylated aptamers were immobilized via a streptavidin–biotin system. A custom-made angle-scanning SPR system based on the Kretschmann configuration was used for measurements. The developed aptasensor had a linear detection range of cTnI from 20 ng/mL to 600 ng/mL and a detection limit of 20 ng/mL. Due to the antifouling property of the zwitterionic peptide, the developed aptasensor had a high resistance to protein fouling.

Long-range surface plasmon-polariton (LRSPP) waveguides were used as biosensors for label-free detection of cTnI [43]. The sensors consist of 5 µm-wide, 35 nm-thick gold stripes embedded in a low-index optical-grade fluoropolymer (CYTOP) with fluidic channels etched to the Au surface of the stripes. Direct and sandwich assays were developed and demonstrated over a concentration range from 1 to 1000 ng/mL, yielding detection limits of 430 pg/mL for the direct assay and 28 pg/mL for the sandwich assay, the latter being physiologically relevant to the early detection or onset of AMI.

**Figure 1:**
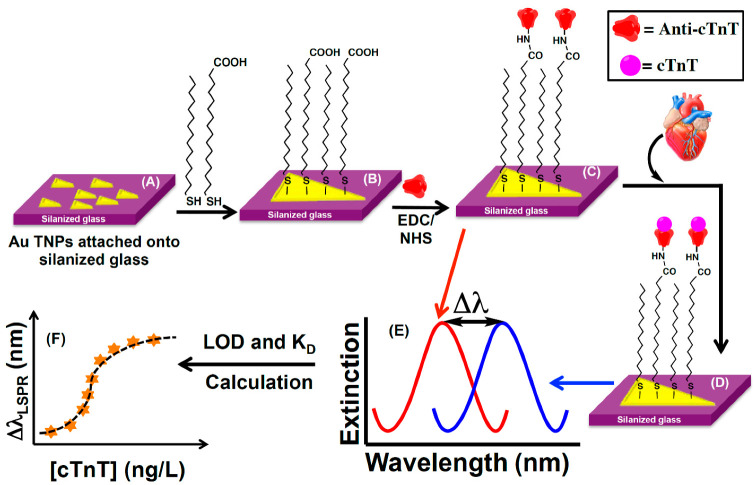
Design of a chip-based format LSPR cTnT biosensor. (**A**) Au TNPs attached to silanized glass, (**B**) after being functionalized with a 1:1 mole ratio of 1-dodecanethiol and 16-mercaptohexadecanoic acid, (**C**) further functionalization with anti-cTnT through EDC/NHS amide coupling to complete the nanosensor, (**D**) detection of cTnT upon binding to anti-cTnT on sensor surface, (**E**) representation of nanosensor absorption maxima (λLSPR) peak shift before and after binding of cTnT, and (**F**) relationship between ∆λLSPR and cTnT concentration to calculate the LOD and KD. For simplicity, only one Au TNP is shown in the functionalization steps. Reproduced from [44] with permission from The Royal Society of Chemistry.

A nanoplasmonic biosensor chip was developed by [44] to assay cardiac troponin T (cTnT) in human biofluids (plasma, serum, and urine) with high specificity. The sensing mechanism is based on the adsorption model that measures the localized surface plasmon resonance (LSPR) wavelength shift of anti-cTnT functionalized gold triangular nanoprisms (Au TNPs) induced by a change in their local dielectric environment upon the binding of cTnT (Figure 1). Controlled manipulation of the sensing volume and decay length of Au TNPs together with the appropriate surface functionalization and immobilization of anti-cTnT onto TNPs allowed the attainment of the limit of detection (LOD) of the cTnT assay at an attomolar concentration (~15 aM) in human plasma.

A biosensor based on a plasmonic exposed core optical fiber tip was developed for the rapid and label-free detection of the N-Terminal portion of the NT-proBNP [45]. The biosensor is based on a fiber tip covered with a gold layer, enabling SPR measurements that were functionalized with anti-NT-proBNP antibodies. It was capable of monitoring NT-proBNP concentrations from 0.01 to 100 ng/mL, in a concentration range of clinical interest [45].

An ultrasensitive SPR immunoassay was developed for the specific detection of human cTnI. Based on the classical thin gold layer as the SPR sensing film, the surface was further modified by hollow gold nanoparticles (HGNPs) and polydopamine (PDA) sequentially and then immobilized with antibodies for specific recognition of the target analyte. The interaction between the localized surface plasmon resonances of HGNPs and the propagating plasmon on the surface of the gold film leads to the amplification of the SPR response signal. For additional sensitivity increases, the sample was incubated with specific magnetic probes made of PDA-wrapped magnetic multi-walled carbon nanotubes (MMWCNTs-PDA) conjugated with detection antibodies (dAb). The magnetically assisted extraction of the target from the sample overcomes the disadvantage of slow diffusion limited mass transfer and matrix interference, reducing the nonspecific interferences while detecting cTnI in human serum. The combination of the above improvements results in the significant sensitivity enhancement of the SPR immunoassay. The concentration of cTnI with minimum detectable SPR response obtained by the assay was 1.25 ng/mL [46].

**Figure 2:**
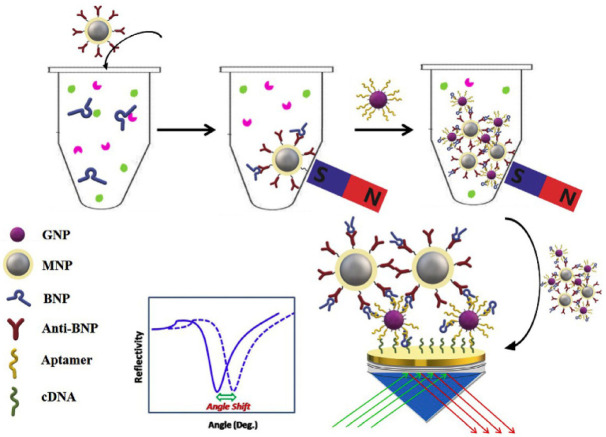
Schematic of the BNP SPR sensing strategy via magnetoplasmonic nanocomposites for signal amplification. The change in refractive index at the gold sensor’s surface is translated into a shift in the resonance angle. More details are given in the text. Reproduced from [47] with permission from Elsevier.

One study led by Zhao [47] achieved the detection of BNP in serum samples using aptamer-functionalized Au nanoparticles (GNPs-Apt) and antibody-modified magnetoplasmonic nanoparticles (MNPs-Ab) for dual evaluation. Both types of nanoparticles (NPs) specifically recognize BNP to form magnetoplasmonic nanoconjugates (NCs). Avoiding degradation is critical for analysis, so applying an external magnetic field makes the separation of the analyte from the complex samples possible. Next, the recognition of NCs is carried out by complementary DNA (cDNA) of the aptamer immobilized on the gold film of an SPR chip (Figure 2). Therefore, the refractive index of the gold surface is significantly modified due to strong electronic coupling between MNPs and the surface plasmon wave of GNPs. The linear range obtained with this method is from 0.1 pg/mL to 100 pg/mL and the limit of detection equals 28.2 fg/mL. The selectivity matter was addressed using some proteins and molecules as interfering substances, bovine hemoglobin (BHb), ascorbic acid (AA), myoglobin (Myo), ovalbumin (OVA) and bovine serum albumin (BSA). Regarding real sample analysis, the group investigated the feasibility of the SPR biosensor in spiked serum. The recoveries were between 92.5 and 113.9%, with RSDs lower than 15%, indicating great accuracy for BNP sensing by the developed SPR method.

In another study by Harpaz [48], an SPR chip was designed and used in a novel point-of-care SPR system for the detection of the stroke biomarkers NT-proBNP and S100β in water and plasma samples. The POC system was based on the commercial PhotonicSys SPR H5 from PhotonicSys (Neveh Shalom-Wahat Alsalam, Israel, www.photonicsys.com, accessed on 1 June 2023). The SPR chip had a bimetallic composition consisting of 30 nm silver and 15 nm gold. The chip was functionalized with thiols and then the specific antibodies for the target biomarkers were immobilized by amino-coupling. NT-proBNP and S100β were detected in a range of clinically relevant concentrations for stroke, from 0.1 ng/mL to 10 ng/mL. In conclusion, SPR-based biosensors are able to detect CVD biomarkers in a label-free and fast manner. Moreover, these biosensors can be implemented in point-of-care (POC) devices due to their versatility, long-term stability, and simple concepts. Amongst the various SPR detection methods, LSPR biosensors proved to be the most sensitive, with an LOD in an attomolar range. Various enrichment techniques (adding affine or magnetic tags) may further improve the detection limit and reduce the nonspecific response of complex clinical samples.

### 3.2. SERS Biosensors for Cardiac Biomarkers

Raman spectroscopy relies on the measurement of the low amounts of inelastically scattered light at specific frequencies, produced as a result of molecular polarizability induced by the vibrations of the chemical bonds and groups of chemical bonds in a molecule under laser irradiation. This non-destructive optical method enables the acquisition of a spectrum whose structure is very specific to a certain molecule as well to the interaction between different molecules; it can therefore provide information on a substance’s identity, polymorphism and crystallinity. Surface-enhanced Raman spectroscopy (SERS) combines outstanding features for sensing applications, such as specific identification and structural information about the molecular species based on their unique vibrational Raman fingerprint, as well as ultrasensitive detection down to a single molecule [49,50]. SERS largely relies on collective oscillations of conduction electrons known as surface plasmon resonances that produce drastic amplification of the electromagnetic fields near the surface of noble-metal nanostructures. These, in turn, significantly enhance the Raman signal from the molecules placed in their close vicinity up to 10^8^–10^10^ orders of magnitude through the so-called electromagnetic mechanism (EM). The molecules directly adsorbed on the nanostructured substrate can experience a charge transfer with the metal surface, leading to additional enhancement of the Raman signal of 10^1^–10^3^ orders of magnitude through the so-called chemical charge transfer (CT) mechanism [49,50,51,52].

The SERS technique has been widely employed as a powerful tool in the development of sensing bioassays for the selective, sensitive and quantitative detection of various cardiac biomarkers. Some of the fabricated SERS bioassays involve the immobilization of the cardiac biomarkers onto nanostructured surfaces, followed by direct analysis and identification of their Raman spectral fingerprint. However, this strategy, called direct SERS detection, suffers from low selectivity due to the multiple components found in the biological samples, which can interfere with the SERS signal of the biomarker of interest. This in turn complicates the data analysis and limits accurate biomarker quantification. Indirect SERS detection has been proposed as an alternative to direct detection to improve the selectivity of the assay and simplify the data analysis. For this, the SERS substrate is modified with Raman reporters and receptors to ensure the specific capture of the target biomarkers. SERS nanotags built on noble-metal nanoparticles conjugated with Raman reporters and specific receptors were also used to increase the selectivity of the assay and accomplish the simultaneous determination of multiple target cardiac biomarkers.

Recent years have witnessed the production of and applications in the sensing of new nanomaterials for enhanced sensitivity of SERS detection, new strategies for the selective and accurate detection in biological samples, in the range relevant for diagnosing CVDs, multiplexed detection and increased use of portable equipment.

For example, nanomaterials such as gold or silver nanoaggregates, core–shell plasmonic bimetallic nanoparticles, hybrid plasmonic–magnetic nanoparticles were coupled with specific bioreceptors (mainly antibodies) and Raman reporters such as rhodamine-6G (R6G), nile blue A (NBA), malachite green isothiocyanate (MGITC), methylene blue (MB), 4-mercaptobenzoic acid (4-MBA), etc., for the selective and ultrasensitive detection of several cardiac biomarkers. Different plasmonic nanoplatforms were fabricated and optimized for high sensitivity for either the direct or indirect SERS detection of various cardiac biomarkers, including CRP [53], cTnI, B-type natriuretic peptide (BNP), CK-MB, Myo, NT-proBNP, neutrophil gelatinase-associated lipocalin (NGAL), glycogen phosphorylase isoenzyme BB (GPBB), neuropeptide Y (NPY) and heart-type fatty acid-binding protein (H-FABP). The following paragraphs discuss several successful SERS biosensors for CVD summarized in Table 3, with emphasis on the nanostructure designs proposed for efficient sensing.

Benford et al. developed the first SERS assay to qualitatively analyze three cardiac biomarkers in diagnosing acute coronary syndrome [54]. Specifically, SERS active aggregated gold nanoparticles (AuNPs) trapped at the entrance of a nanofluidic device were used as sensing elements to detect BNP, cTnI, and CRP. This sensing platform enables the detection and identification of BNP, cTnI, and CRP at physiologically relevant concentrations. Unfortunately, no real sample analysis was reported in the work. A key issue in SERS-based detection bioassays represents the biosensor’s capacity to detect the target analyte with high specificity. Given this, the same group designed an improved SERS bioassay for the specific detection of CRP [55]. The proposed platform incorporates agarose beads functionalized with an anti-CRP antibody for the specific capture of CRP, aggregated gold nanoparticles as the SERS units, and CRP labeled Rhodamine-6G (R6G) as a target analyte. Besides the specific detection of CRP, a correlation between the amount of CRP and the SERS signal of R6G was also observed. However, there was no validation of the results in clinical samples.

Knowing the concentration of cardiac biomarkers in human blood is essential in diagnosing cardiovascular diseases. Therefore, the efforts in designing SERS assays were focused not only on selective detection but also on the quantification of the biomarkers in blood samples. A good example is a study reported by Cong et al. [56]. In their work, the SERS technique and an enzyme catalysis bioassay (ELISA) were combined for selective and sensitive detection and quantification of the human cardiac isoform of troponin T, cTnT in human serum. The proposed detection strategy involves the use of citrate-capped spherical AuNPs as a SERS substrate, and the resulting product of the enzyme-catalyzed 3,3′,5,5′-tetramethylbenzidine reaction, TMB^2+^ as a SERS probe.

Remarkably, the developed biosensor achieved a broader linear concentration range (2~320 pg/mL) and an improved sensitivity (limit of detection-LOD of 2 pg/mL) compared with the UV-Vis technique (linear concentration range 4~80 pg/mL and LOD of 4 pg/mL). The performance of the sensor assay for clinical applications was evaluated with two serum samples containing two concentrations of cTnT, 16 and 8 pg/mL, respectively. The relative standard deviation for these two concentrations was 0.017 and 0.093 and the average recoveries were 100.01% and 86.815%, respectively. Coté and co-authors also exploited AuNPs to develop an optofluidic device for SERS detection of myoglobin [57]. The fabricated device comprising plastic plates, rubber layers, and a nanoporous membrane was exposed to a mixture containing rhodamine-6G (R6G) labeled myoglobin and colloidal AuNPs. The aggregation of AuNPs on the nanoporous membrane led to the formation of a robust, sensitive and reproducible SERS active plasmonic substrate. The intensity variation of a characteristic Raman band of R6G was used to quantify myoglobin concentration in solution over a physiologically relevant range (1.2 nM to 30 nM). To further evaluate the performance of the assay in complex samples, bovine serum albumin (BSA) was introduced as a possible interfering compound. Unfortunately, a decrease in the SERS signal was noticed in the presence of BSA for all concentrations of myoglobin tested. Furthermore, no real sample analysis was provided in the work. Later, silver nanoaggregates were exploited by the same authors to fabricate a bioassay for SERS detection of the human cardiac Troponin I (cTnI) in solution [78]. In this system, silver nanoparticles (AgNPs) were first encoded with the Raman reporter molecule 5,5-dithiobis-(2-nitrobenzoic acid (DTNB) and then aggregated to give a strong and stable SERS signal. In the second step, the Ag nanoaggregates were encapsulated in a silica shell to stabilize them and facilitate their further bioconjugation. Finally, the core–shell Ag nanoaggregate–silica architecture was coated with polyethylene glycol (PEG) and functionalized with cTnI protein and BSA to endow them with an affinity for the cTnI antibody. Unfortunately, this study did not address the detection of cTnI and instead only examined the SERS signal of the nanoconjugate. Three-dimensional silver anisotropic nano-pinetree array modified indium tin oxide (Ag NPT/ITO) was proposed by El-Said and co-authors as an alternative to silver nanoaggregates to develop an ultrasensitive SERS platform for the direct, label-free detection of myoglobin [58]. Another three Ag nanostructures/ITO substrates (Ag nanoaggregates/ITO, Ag nanorods/ITO and Ag nanobranched/ITO) were also prepared and compared with Ag NPT/ITO regarding their SERS performance. The fabricated Ag NPT/ITO substrate showed the best Raman signals, yielding an LOD of 10 × 10^−9^ g/mL and a wide working range for myoglobin quantification from 5 × 10^−6^ to 10 ng/mL. The sensor performance for clinical applications was evaluated by analyzing urine samples spiked with a known amount of myoglobin. A linear relationship between the Raman intensity and the myoglobin concentration in urine over a range of 10 ng/mL to 5 μg/mL was obtained. In addition, the calibration curves for urine and buffer have almost the same slopes, demonstrating the accuracy of the detection without interference. However, there was no validation of the results by parallel analysis using a standard method.

Ultrasensitivity and high specificity were also reported by Gao et al., who managed to design a novel hybrid microfluidic chip for simultaneous SERS detection of CK-MB and cTnI cardiac markers [59]. In this system, a SERS substrate fabricated by in situ synthesis of AuNPs on the patterned paper microchannels was used as a capture platform, while AuNPs labeled with malachite green isothiocyanate (MGITC) Raman reporter molecules were employed as SERS detection nanotags. To achieve specificity toward CK-MB and cTnI, the SERS platform and the Raman probes were conjugated with capture and detection antibodies against CK-MB and cTnI. Selective quantification of CK-MB and cTnI was accomplished by measuring the SERS signal on a sandwich-type nanoarchitecture formed after the immune reaction, yielding an LOD of 7.92 pg mL^−1^ and 2.94 pg mL^−1^ for CK-MB and cTnI, respectively. However, some interfering SERS signal was noticed in the presence of BSA, thrombin, and PSA spiked in serum samples due to the non-specific adsorption of SERS nanotags on the detection area.

Core–shell plasmonic bimetallic nanoparticles have also been employed in the development of various SERS bioassays as they offer several advantages compared with monometallic nanoparticles, such as high stability and reproducibility of the Raman signal, as well as an increased SERS performance. Both Raman reporter-labeled and reporter-embedded core–shell nanotags were prepared and exploited as ultrasensitive SERS nanoprobes for the selective determination of cardiac biomarkers.

For instance, Bai et al. fabricated three classes of bimetallic core–shell SERS nanoprobes and one class of monometallic nanoprobe and investigated their SERS activity by experimental measurement and theoretical analysis [60]. The monometallic class was built on citrate-capped AuNPs encoded with Nile blue A (NBA) dye. Two classes of core–shell nanoprobes consist of a metallic core (Au or Ag) with a metallic shell (Ag or Au) and NBA embedded at their interface and also labelled at their surface. The other class of core–shell nanoprobes was built on Au-core labeled with NBA, a silver shell labeled with NBA and then etched with HAuCl_4_ to form Au@AgAuNPs with nanometric gaps inside. The obtained Au@Ag-Au NPs were further encoded with NBA. Both experimental and theoretical results showed that Au@Ag-Au NPs exhibited the best SERS performance due to the strong electromagnetic field created in the nanogaps between the core and shell. Therefore, the authors selected Au@Ag-Au NPs to develop SERS-based lateral flow assay strips for selective, highly sensitive and quantitative analysis of cardiac troponin I (cTnI). To achieve specificity toward cTnI, the fabricated Au@Ag-AuNPs and test line were conjugated with detection and capture antibodies, respectively. The quantification of cTnI was performed by monitoring the SERS intensity of a characteristic Raman band of NBA in a sandwich immunocomplex formed after the exposure of the sample pad to various concentrations of cTnI. The designed SERS-based lateral flow assay strips provide reproducible, selective and highly sensitive detection of cTnI with the LOD of 0.09 ng mL^−1^. Even though no interference signal was found when CRP, BNP, Myo, and CK-MB were present, real sample analysis was unfortunately not addressed in this study.

Zhang et al. also exploited core–shell SERS nanotags to develop SERS-based lateral flow assay strips for the simultaneous detection of Myo, cTnI and CK-MB on three test lines [61]. In their design, SERS nanotags were built on an Ag core with an Au shell and NBA Raman reporter molecules embedded at their interface. The fabricated SERS nanotags were then conjugated with detection antibodies of three biomarkers to form an immunocomplex with the capture antibodies on a nitrocellulose membrane when the target biomarkers were present. The intensity of the SERS spectra recorded from the three test lines at 785 nm laser excitation was used for quantitative analysis of Myo, cTnI, and CK-MB. The designed SERS-based lateral flow assay provides reproducible, highly sensitive and multiplex detection of Myo, cTnI and CK-MB with a wide linear dynamic range and the LODs of 3.2, 0.44, and 0.55 pg/mL, respectively. The diagnostic performance of the assay was evaluated with 50 serum samples collected from hospitalized patients suffering from AMI and the results were compared with the FDA-approved clinical chemiluminescence immunoassay (CLIA) method. Passing–Boblok regression and Spearman’s rank correlation coefficient were used to examine the linear dependence between the two methods. A good linear correlation between the two methods was obtained. The assay has better sensitivity than CLIA, is low-cost and easy to use and requires 15 min per marker. However, the developed sensor has speed constraints, necessitating the examination of three test lines. Thus, shortly thereafter, the same group reported an improved version of the SERS-based lateral flow assay for rapid, multiplex quantitative detection of CK-MB, cTnI, and Myo using a single test line [62]. In their design, three different SERS nanotags, namely methylene blue (MB), nile blue A (NBA) and rhodamine 6 G (R6 G), were built on an Ag core with an Au shell and Raman reporters embedded at their interface. The SERS spectra recorded from a single test line at 785 nm laser excitation show distinct features for all corresponding nanotags of biomarkers. The designed sensor yielded an LOD of 0.93, 0.89, and 4.2 pg/mL and linear dynamic range of 0.02−90, 0.01−50, and 0.01−500 ng/mL for CK-MB, cTnI, and Myo, respectively. Five human serum samples from hospitalized patients with AMI were examined to ascertain whether the assay is suitable for usage in clinical settings. The CLIA approach was used to compare the outcomes. Real samples yielded very good recoveries, ranging from 86.7% to 113.5%, demonstrating the accuracy of the assay.

Yu et al. also developed a core–shell SERS nanotag-based sandwich immunoassay for rapid, sensitive and simultaneous detection of cTnI and CK-MB [63]. The sandwich system was based on Au@Ag core–shell nanoparticles conjugated with malachite green isothiocyanate (MGITC) and polyclonal antibodies as the SERS detection element and a gold-patterned chip functionalized with monoclonal antibodies as the SERS active template. The designed assay enabled quantitative analysis of cTnI and CK-MB with an LOD of 8.9 pg/mL and 9.7 pg/mL for cTnI and CK-MB, respectively. The clinical applicability of the sensor was evaluated with five serum samples collected from patients with AMI and the results were compared with those obtained with a commercially available chemiluminescence assay. The concentrations of cTnI and CK-MB determined by the SERS-based assay were comparable to those determined by the chemiluminescence technique, and they were all within the clinically acceptable range. Another approach for ultrasensitive simultaneous detection of cTnI, N-terminal prohormone of brain natriuretic peptide (NT-proBNP) and neutrophil gelatinase-associated lipocalin (NGAL), was reported by Zhu and co-authors [64]. Their strategy involves using bimetallic Ag-Au nanostars conjugated with Raman reporters (4-mercaptobenzoic acid (4-MBA), 5′-dithiobis-(2-nitrobenzoic acid) (DTNB), 2-naphthalenethiol (NT)) and detection antibodies as SERS nanotags and a three-dimensional ordered macroporous Au-Ag-Au plasmonic array conjugated with capture antibodies as a substrate to improve the reproducibility and sensitivity of the assay. The sensitivity of the system is achieved through the formation of Raman hot-spots between the nanotags and substrate after biomolecular recognition. This SERS-based sandwich immunoassay allowed for sensitive and reproducible multiplex detection, yielding an LOD of 0.76, 0.53 and 0.41 fg/mL for cTnI, NT-proBNP and NGAL, respectively. The suitability of the developed immunoassay for clinical applications was also demonstrated by the simultaneous determination of cTnI, NT-proBNP and NGAL in human serum samples. The results were compared to those obtained using the dot immunogold filtration test (DIGFA) to confirm the accuracy of the immunoassay. The two techniques’ agreement was found to be reasonable, thus proving the potential of the proposed sensor for clinical applications.

**Figure 3:**
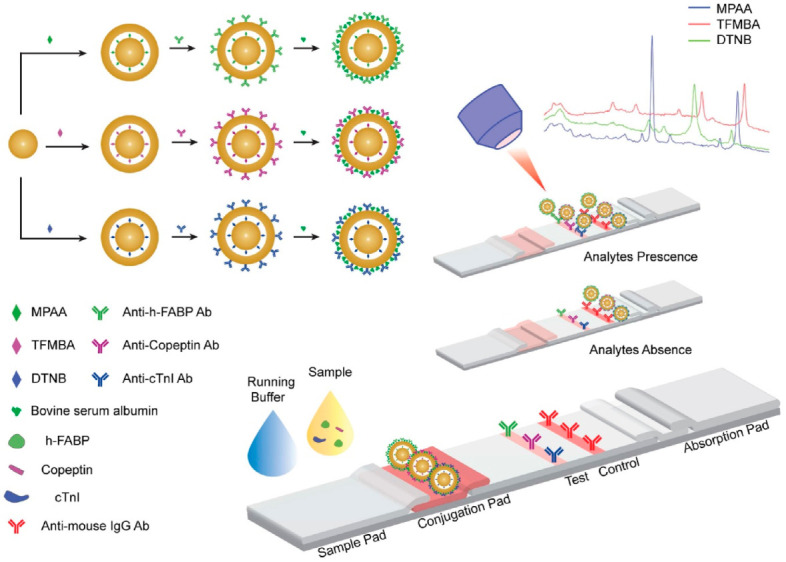
Schematic illustration of the process to prepare antibody conjugated gap-enhanced nanoparticle, and the lateral flow strip for a multiplex detection of the biomarker panel for myocardial infarction. Reprinted with permission from [66]. Copyright (2022) Elsevier.

Raman reporter-embedded Au nanorod-core Au-shell nanotags were employed by Khlebtsov et al. for the design of a SERS-based lateral flow immunoassay for fast, sensitive, semiquantitative determination of cTnI [65]. Detection of cTnI was performed by Raman mapping of the test zone, while quantification was achieved by monitoring the SERS intensity of a specific band of 1,4-nitrobenzenthiole (NBT) Raman reporter following the exposure of NPs to different concentrations of cTnI, reaching an LOD of 0.1 ng/mL. However, the selectivity of the assay and analysis of the real samples were not reported in this work. Going one step forward, Tu et al. recently employed gap-enhanced nanoparticles (GeNPs) as ultrasensitive SERS nanotags to design a paper-based immunoassay for the simultaneous quantification of three myocardial infarction biomarkers: cardiac troponin I (cTnI), copeptin, and heart-type fatty acid-binding protein (h-FABP) [66]. As schematically illustrated in Figure 3, GeNPs consist of three different SERS tags (4-mercaptophenylacetic acid (MPAA), 2,3,5,6-tetrafluoro-4-mercaptobenzoic acid (TFMBA) and 5,50-dithiobis (2-nitrobenzoic acid) (DTNB)) built on Raman reporter-embedded gold-core gold-shell with nanometric-size gaps of 0.9–1.1 nm created at the interface of the core–shell nanoarchitecture.

Due to the high electromagnetic enhancement of the Raman signal inside the narrow nanogaps, GeNPs enabled an increase in the SERS signal by 105–250 times compared to the Au core. To achieve specificity toward the three target biomarkers, the fabricated GeNPs-based SERS nanotags were conjugated with detection antibodies against cTnI, copeptin and h-FABP (Figure 3). A single test line consisting of a nitrocellulose membrane conjugated with capture antibodies specific to cTnI, copeptin and h-FABP was used. A sandwich immunocomplex was formed on the test line only when the target biomarkers were present. The SERS spectra recorded from the test line at 780 nm laser excitation exhibited distinct SERS features related to each target biomarker, thus allowing the simultaneous detection, spectral discrimination and quantification of cTnI, copeptin and h-FABP with an LOD of 0.01 ng/mL, 0.86 ng/mL, 0.004 ngmL for cTnI, h-FABP, and copeptin, respectively. Notably, the developed paper-based SERS assay enabled the quantification of the three biomarkers in human serum samples in a clinically relevant range of concentrations. However, the sensor has some limitations due to the non-specific binding between the three different GeNP/antibody particles, biomarkers, and primary antibodies, which leads to some interference in the multiplex immunoassay.

Even though core–shell bimetallic (Au@Ag or Ag@Au) nanoparticles were extensively employed in several types of SERS bioassays, Tu et al. proposed gold-core silica-shell nanoparticles as SERS nanotags to design a stable, reproducible and cost-effective aptamer-based sandwich assay on a paper strip for the determination of cTnI via surface-enhanced resonance Raman spectroscopy (SERRS) [67]. In the developed design, spherical AuNPs with a diameter of 60 nm were first encoded with the Raman reporter molecule malachite green isothiocyanate (MGITC) and then encapsulated in a silica shell. For specific recognition of cTnI, the core–shell nanoparticles were functionalized with a secondary aptamer of cTnI, while the test line was modified with a primary aptamer of cTnI. The specific molecular interaction of aptamers and cTnI results in a sandwich architecture between the core–shell nanoparticles and the test line. Selective and sensitive detection of cTnI was accomplished by measuring the SERRS signal of the test line under resonant excitation at 638 nm, reaching a detection range of 0.016 to 0.1 ng/mL, with an LOD of 0.016 ng/mL. The suitability of the developed aptamer-based paper strip assay for clinical applications was demonstrated by SERRS detection and quantification of cTnI in serum samples. Recovery factors were evaluated by spiking cTnI at 0.03 and 0.05 ng∕mL in human serum without any treatment. The obtained values, in the range of 93.8% to 95.8%. indicated the high accuracy of the method. Furthermore, the aptamer-based paper strip assay showed high stability after 10 days of storage at room temperature. However, the assay remains to be confirmed by parallel analysis with a larger sample set and a standardized technique.

Raman-encoded gold or silver nanoparticles enveloped in a silica shell were also employed by Lim et al. as extrinsic SERS probes to design a microfluidic paper-based device (μPAD) for simultaneous quantitative SERS measurement of three cardiac biomarkers: glycogen phosphorylase isoenzyme BB (GPBB), cTnI and creatine kinase-MB isoenzymes (CK-MB) [68]. The proposed bioassay ensured high reproducibility and ultrasensitivity to detect the three cardiac biomarkers with an LOD of 8, 10, and 1 pg/mL for GPBB, CK-MB and cTnT, respectively, showing high accuracy even in the detection of clinical samples of human serum. The results were further validated by a laboratory reference method, Siemens Centaur XPT Immunoassay System. Additionally, a predictive model was created to estimate the unknown concentration of cardiac biomarkers in serum samples and eliminate the standard calibration curves. A good fit of the predicted and experimental data was obtained.

Combining plasmonic substrates with magnetic nanoparticles has brought new advantages in SERS detection of cardiac biomarkers, enabling an increased sensitivity and a simple analysis procedure after magnetic separation. For example, a fast and sensitive SERS-based competitive immunoassay for the simultaneous detection of cTnI and CK-MB was demonstrated by Choo et al. [69]. Their method involves the use of magnetic beads functionalized with monoclonal antibodies for cTnI and CK-MB as capture agents and two different SERS nanoprobes based on hollow gold nanospheres conjugated with Raman reporters and cTnI and CK-MB antigens as SERS sensitive platforms for cTnI and CK-MB recognition. The proposed detection strategy involves the competitive interaction of free target antigens and antigen-conjugated SERS nanoprobes with monoclonal antibodies on magnetic beads, followed by magnetic separation and SERS analysis of the supernatant. The results showed that the developed SERS-based competitive immunoassay achieves a 100 to 1000-fold increase in sensitivity compared to electro-chemiluminescent assay, yielding an LOD of 42.5 pg/mL and 33.7 pg/mL for CK-MB and cTnI, respectively. The agreement of the two analytical methods was validated with Bland–Altman analysis and Passing–Bablok regression analysis. Remarkably, the fabricated SERS-based competitive immunoassay enables simultaneous quantitative detection of cTnI and CK-MB in patient serum at a single excitation wavelength.

Another study used metal–organic frameworks (MOFs)@Au tetrapods (AuTPs) immobilizing toluidine blue as the SERS tag and Au nanoparticles functionalized CoFe_2_O_4_ magnetic nanospheres (CoFe_2_O_4_@AuNPs) as the purification and signal amplification agents to develop a highly efficient SERS-based sandwich immunosensor for ultrasensitive detection of N-terminal pro-brain natriuretic peptide (NT-proBNP) 70]. The fabricated SERS immunosensor enables specific capture of NT-proBNP via antigen–antibody immunoreaction, yielding a wide linear range for NT-proBNP quantification from 1 fg/mL to 1 ng/L and an LOD of 0.75 fg/mL. Good recovery factors, in the range of 90.66% to 105.1%, were obtained in human real serum samples, confirming the accuracy of the method. However, the results remain to be verified through parallel analysis with a standard commercially available technique.

Recently, Zheng et al. also employed CoFe_2_O_4_@AuNPs conjugated with specific antibodies to ensure the magnetic purification of the analytes and improve the sensitivity of their microfluidic immunosensor developed for specific SERS detection of the brain natriuretic peptide (BNP) cardiac biomarker [71]. Apart from CoFe2O4@AuNPs, a SERS substrate consisting of an metal–organic framework and Au-HEPES coupling nanoparticles (AuHPs) conjugated with toluidine blue and antibody against BNP was also introduced to further enhance the Raman signal of the sensor. Specific quantitative detection of BNP was performed by measuring the SERS signal with a portable Raman spectrometer based on a sandwich approach. The proposed immunosensor exhibited high stability, portability and ultrasensitivity, yielding an LOD at a level of pg mL^−1^. However, the performance of the immunoassay in clinical samples was not reported in the study. A highly sensitive SERS-based immunoassay for selective quantitative detection of cTnI has been demonstrated by Fu and co-authors [72]. The fabricated immunosensor is composed of antibody/Raman reporter conjugated AuNP–functionalized graphene oxide as the SERS nanotags and signal amplification elements and antibody-modified magnetic beads as the capture probe and separation agents. By exploiting the high SERS performance of graphene oxide/AuNPs and strong binding chance between cTnI and the GO/AuNP, an ultrasensitive analysis of cTnI is achieved with the LOD of 5 pg/mL^1^ and a linear range of detection from 0.01 to 1000 ng/mL. The suitability of the designed SERS immunoassay for practical applications was also demonstrated by the determination of TnI in serum substitute media. However, the results remain to be confirmed in clinical samples and compared with those obtained with a standard technique.

Wen et al. demonstrated an innovative, portable reusable accurate diagnostics (PRADA) SERS-based immunoassay for simultaneous quantitative detection of troponin I (cTnI) and neuropeptide Y (NPY) in a microfluidic platform [73].

**Figure 4:**
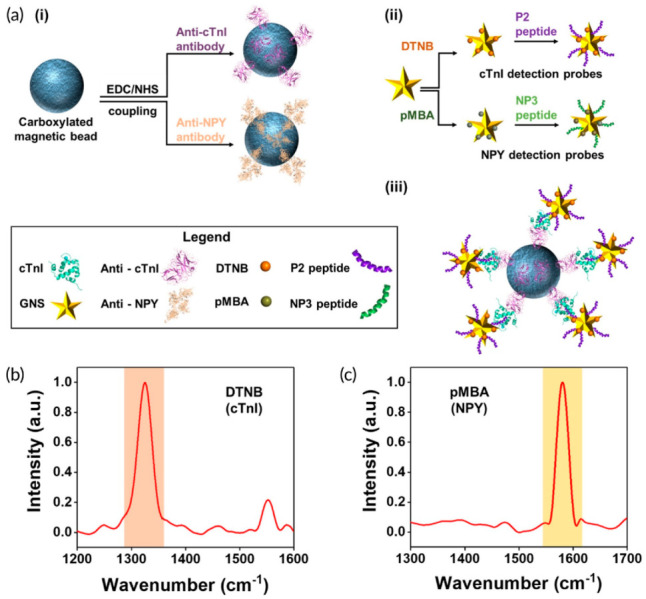
Fabrication of PRADA. (**a**) Schematic of the synthesis of capture and detection probes. (**i**) Magnetic beads functionalized with pAbs as capture probes. (**ii**) GNSs conjugated with SERS barcodes and peptide BREs as detection probes. (**iii**) The representative complete immunocomplex formed by capture probes, target antigens, and detection probes. (**b**,**c**) Normalized Raman spectra of GNSs functionalized with DTNB (1325 cm^−1^) and pMBA (1580 cm^−1^) for cTnI and NPY detection, respectively; the signature peaks are highlighted. BREs, biorecognition elements; GNSs, gold nanostars; pAbs, polyclonal antibodies; PRADA, portable reusable accurate diagnostics with nanostar antennas; SERS, surface-enhanced Raman spectroscopy. Reprinted with permission from [73]. Copyright (2020) John Wiley & Son.

As illustrated in Figure 4a, antibody-conjugated magnetic beads were used as the capture platform, while SERS nanotags based on gold nanostars labeled with two different Raman reporters and peptide biorecognition elements were introduced as SERS detection probes. The SERS spectra recorded from the sandwich-type-immunocomplex at 785 nm laser excitation show the distinct features of the two nanotags corresponding to cTnI and NPY (Figure 4b). Specifically, the SERS intensity of DTNB at 1325 cm^−1^ was used for cTnI quantification, while NPY determination was accomplished by monitoring the SERS signal of pMBA at 1580 cm^−1^. The PRADA sensing device exhibited high sensitivity, achieving an LOD comparable with the commercially available test kits: 0.0055 ng mL^−1^ and 0.12 ng mL^−1^ for cTnI NPY, respectively. Moreover, the sensor chip can be regenerated, thus being reusable for multiple detection cycles. Remarkably, the developed PRADA assay showed high accuracy and reproducibility in evaluating the cTnI in cardiac patient serum, achieving a limit of quantification (LOQ) of ≥0.03 ng/mL, which is comparable to commercial assays and lower than many troponin immunoassays reported in the literature. Furthermore, the clinical performance of the PRADA assay was validated by parallel measurements with the ABBOTT ARCHITECT chemiluminescence assay system and Passing–Bablok regression analysis.

Sandwich-based immunoassays are constantly developed for SERS quantification of cardiac biomarkers. For instance, a new SERS-based magnetic immunoassay was designed by Hu et al., which combined magnetic beads and Raman-reported embedded Au-core Ag-shell nanotags for sensitive and selective determination of cTnI [74]. In this system, the Raman-embedded core–shell nanotags were introduced to increase the stability and sensitivity of the SERS sensor, while magnetic beads ensured the magnetic separation and concentration. Specific recognition of cTnI was accomplished by modifying both SERS tags and magnetic beads with antibodies against cTnI. Once the sandwich-type was formed based on an immune reaction, it was magnetically separated and subjected to SERS analysis using a portable Raman instrument. The developed immunoassay achieved an LOD of 9.80 pg/mL. Fifty serum samples from AMI patients were analyzed using the SERS assay and the FDA-approved clinical chemiluminescence immunoassay (CLIA) to assess the clinical performance of the proposed sensor and its diagnostic potential. The two approaches were strongly correlated, demonstrating the practical usability of the SERS-based immunoassay. This kind of immunoassay was also demonstrated by the same group of authors for selective, sensitive, accurate simultaneous determination of cTnI and heart-type fatty acid-binding protein (H-FABP) [75]. The obtained LOD was 0.6396 and 0.0044 ng/mL for H-FABP and cTnI, respectively, which is much lower than the clinical cutoff value for the diagnosis of acute myocardial infarction disease. Moreover, the described approach allowed simultaneous determination of H-FABP and cTnI in human serum samples, demonstrating great potential for clinical applicability. High recovery factors in the range of 96.4–110.0% were obtained, indicating the accuracy of the method. However, cTnI and H-FABP were spiked in already diluted serum samples of healthy people. Results remain to be confirmed in undiluted samples collected from AMI patients.

Atomically flat Au nanoplates present great potential as sensing nanoplatforms as Lee et al. exploited such substrates to design an innovative sandwich-based approach for SERS detection of cTnI at the attomolar level [76]. In this strategy, AuNPs conjugated with a cTnI aptamer modified with a Raman reporter molecule served as a SERS detection probe, while the aptamer-conjugated Au nanoplates enabled the specific capture of cTnI. The detection of cTnI was accomplished by measuring the SERS signal of the AuNPs-on-nanoplate architecture formed upon the specific capture of cTnI. By optimizing the immobilization of the aptamer onto Au nanoplate, the binding capacity for cTnI was significantly improved. Therefore, the LOD was determined to be 100 aM (2.4 fg mL^−1^) in buffer solution and 100 fM (2.4 pg m^−1^) in serum samples, respectively, which is much lower than the existing cutoff values. Nine clinical samples from both healthy humans and AMI patients were collected and analyzed in parallel using the developed SERS assay and ELISA. Remarkably, the accuracy of the proposed strategy for cTnI detection is higher than that of the conventional ELISA.

A microcavity-based sandwich immunosensor was designed by Wang et al., who combined the light confinement effect of polystyrene (PS) microcavities and the localized surface plasmon resonance (LSPR) properties of AuNPs to achieve simultaneous ultrasensitive SERS quantification of cTnI and CK-MB [77]. The sensor is composed of PS microspheres modified with AuNPs deposited on a silicon wafer as a capture substrate, and Raman-encoded AuNPs as SERS signal probes, respectively. For recognition of cTnI and CK-MB, both SERS tags and the capture substrate were modified with antibodies specific to cTnI and CK-MB. Sensitive and selective detection of cTnI and CK-MB was accomplished by SERS measurement based on a sandwich strategy, reaching an LOD of 3.16 pg mL^−1^ and 4.27 pg mL^−1^ for cTnI and CKMB, respectively. The performance of the method was evaluated in whole blood samples. The obtained recovery factors of cTnI and CK-MB ranged from 94.9% to 121.6% while the average coefficient of variance (CV (%)) between replicates was below 15%, all of these indicating the high accuracy, reproducibility and stability of the developed SERS immunoassay.

In summary, recent studies concerning SERS-based detection of cardiac biomarkers were focused on sensitivity, selectivity, accuracy, rapidity and portability. Significant attention has also been paid to the multiplexed detection of multiple CVD biomarkers, as shown in Table 3. Most of the reported sensors achieved comparable or superior performance to existing analytical methods such as ELISA, clinical chemiluminescence immunoassay (CLIA), ABBOTT ARCHITECT chemiluminescence assay, Siemens Centaur XPT Immunoassay System, etc. (Table 1), in terms of sensitivity and rapidity. While the above-described detection approaches picture a rich toolbox available to a scientist wishing to develop new methods for CVD biomarkers detection, many studies are at a preliminary level. Further work is necessary to bring these sensors closer to application, to characterize and validate them fully and to apply them on larger sets of clinical samples in order to prove their utility. Increased application of chemometrics and the use of machine learning/AI to interpret the complex SERS spectra and extract the specific information pertaining to CVD biomarkers can substantially and rapidly improve the performance of SERS-based detection methods. Aptamers and MIPs still appear to be underrepresented among the specific receptors used in cardiac biosensors based on SERS. Considering the effervescent research in the field of aptasensors and MIPs for cardiac biomarkers with improved stability and high specificity and reproducibility compared to antibodies, it is anticipated that the number of detection strategies based on the combination of these receptors with SERS will significantly grow in the future.

### 3.3. Fluorescence-Based Biosensors

Fluorescence is a type of photoluminescence and refers to the phenomenon of emitting light, displayed by some atoms and molecules upon reverting back to the ground state after being excited by illumination. Fluorescence is a preferred detection mode in biomarker analysis [79], enabling high sensitivity and multiplexing as probes labeled with various fluorescent dyes or nanomaterials become increasingly available and diversified. Examples of fluorescent probes employed in CVD biomarker detection include fluorochrome dyes and nanomaterials such as lanthanide-doped upconversion nanoparticles (UCNPs) [80], quantum dots (QDs) [81], carbon dots (CDs) [82], europium chelate-contained silica nanoparticle (EuSiNP) [83], metal–organic frameworks, MOFs, [84], etc. These probes have been linked to antibodies [80], aptamers [85] or molecular imprinted polymers (MIPs) [86].

Fluorophore-marked bioreceptors were used in both homogenous and heterogeneous systems (Table 4), selectively binding the analyte and thus enabling the correlation of its concentration with the fluorescence signal.

Among the various types of tests for the fluorescence-based detection of CVDs, lateral flow assays (LFAs) were a preferred choice and appear particularly promising and when designing as they facilitate simple, rapid, low-cost, portable and user-friendly tests [80].

**Figure 5:**
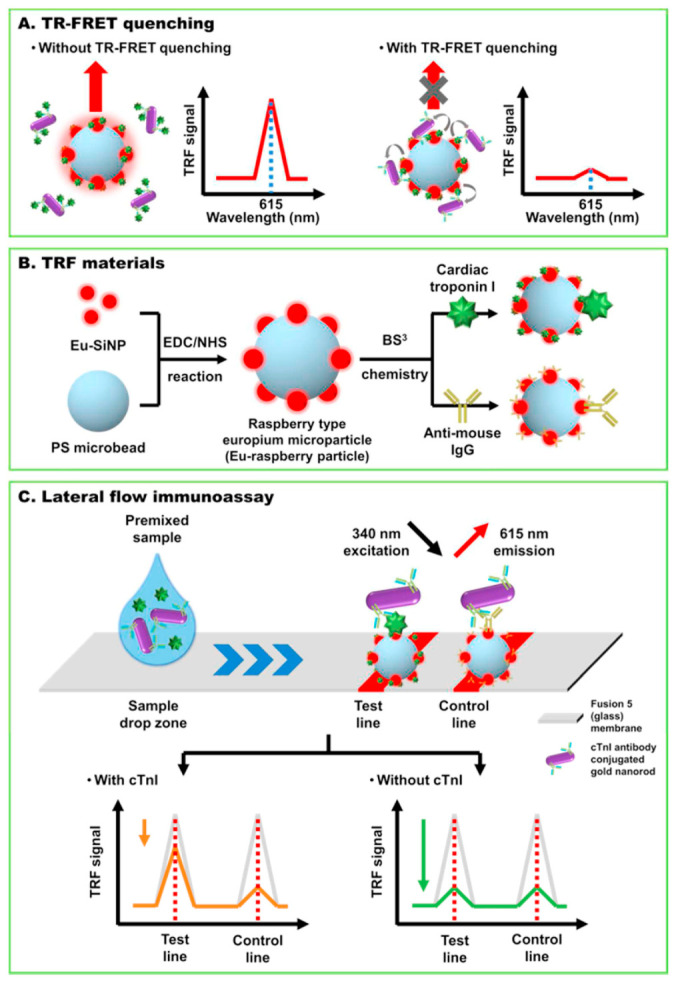
Principle of the TR-FRET LFIA for cTnI. (**A**): Fluorescence quenching mechanism by TR-FRET using Eu-SiNP and GNR. (**B**): The synthesis of the donor raspberry-type Eu-SiNP. (**C**): The competitive assay used in the LFIA: cTnI in the sample competes with cTnI anchored on the Eu-SiNPs at the test line for binding to the cTnI Ab-GNR conjugates. Thus, in the presence of cTnI, the fluorescence at the test line is high. In the absence of the biomarker, the binding of the cTnI Ab-GNR conjugates to the cTni/Eu-SiNPs, drastically quenching the signal at the test line. Reproduced from [83] with permission from Elsevier.

In one example, troponin I detection was achieved by time-resolved fluorescence resonance energy transfer (TR-FRET) using raspberry-type polystyrene microparticles coated with europium chelate-modified silica nanoparticles (EuSiNP) as donor and gold nanorods (GNR) as a fluorescence acceptor [83]. The use of a fusion 5 membrane (a proprietary, commercially available porous and hydrophilic membrane) enabled a simplified lateral flow assay system lacking the sample, conjugate and absorbent pads needed with conventional strips made of nitrocellulose. The test was conceived as a competitive immunoassay whose sensitivity was enhanced thanks to TRF measurements, enabling the removal of background fluorescence by taking advantage of the long fluorescence decay time of lanthanides. The lateral flow strip has a test line consisting of cTnI conjugated Eu-raspberry particles and a control line with immobilized anti-mouse-antibody-conjugated Eu-raspberry particles (Figure 5). The quencher particles, GNR conjugated with a specific antibody for cTnI Ab, are first mixed with the sample. When placed on the designed sample zone of the strip, they advance through capillary force towards the test and control lines. In the absence of cTnI in the sample, the GNR-cTnI Ab particles bind to the cTnI-EuSiNP particles in the test line, drastically decreasing their fluorescence signal. The excess quencher particles are captured at the control line. In the presence of cTnI, the GNR-cTnI Ab bind to the biomarker in the sample instead of the cTnI at the test line; thus, the quenching of the fluorescence signal at the test line is reduced (i.e., the fluorescence is higher). The competitive test was conducted both in buffer and serum samples, featuring LODs of 24 pg/mL and 97 pg/mL, respectively. This LFIA system was compared with a standard cTnI ELISA assay and showed good accuracy.

The traditional bench-top detection equipment for fluorescence measurements is bulky and expensive. Portable readers facilitate the adoption of fluorescence methods by users and fully exploit the potential of LFIA systems for POC measurements. A POC platform including a portable detection module and a sample processing module (LFA strip) relied on upconversion nanoparticles (UCNPs) for myoglobin detection [80]. The UCNPs were NaYF4:Yb, Er@NaLuF4 core–shell nanoparticles, and the specificity of the detection was ensured by a classic sandwich immunoassay. The ratiometric approach, where the concentration of myoglobin was proportional to the fluorescence intensity ratio of test and control (T/C) lines, helped minimize the sensitivity of the assay to possible deviations between different strips. A 10 min period was optimum for the immune reaction to take place so that the T/C ratio reached saturation. The test is rapid, the time per assay being three times shorter than for the standard method. Interestingly, different types of plasma sample (hemolysis, high-bilirubin, and high-lipids) were analyzed to test potential interfering effects. Based on recoveries of 89.0–110.5% from spiked samples and CVs of less than 10%, the authors concluded that the type of sample had little effect on the test result. The results obtained with this LFIA method were compared to the Abbott Chemiluminescence assay, typically used in clinical practice, showing great consistency between them (i.e., coefficient of determination of 0.95, slope of the linear regression = 0.92). Intra and inter-assays performed with LFIA were characterized by coefficients of variation (CVs) under 14%, showing the good precision of the proposed sensor strip.

Molecularly imprinted polymers are increasingly being researched as antibody replacers with the aim to decrease costs and improve the stability and reproducibility of bioanalytical testing devices. Moreover, significant advances have been made with regard to such “plastic antibodies” with high affinity and specificity for high-molecular-weight molecules including protein biomarkers. Due to their large surface displaying a variety of functional groups, developing MIPs with cavities complementary to such large target molecules is a very difficult task.

**Figure 6:**
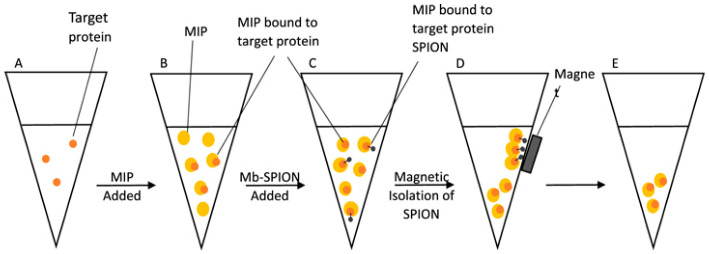
Principle of the MIP-based fluorimetric homogeneous assay for myoglobin. The sample containing myoglobin (**A**) is incubated with fluorescein-tagged MIP (**B**) which binds myoglobin. Next, myoglobin-functionalised SPION particles (“Mb-SPION”) are added to bind the excess MIP (**C**). The SPION particles and conjugates with MIPs are removed via a magnet (**D**). The fluorescence due to the remaining MIP particles (**E**) is finally measured and correlated with the concentration of myoglobin in the sample. Reproduced from [86] with permission from the authors.

An MIP-based fluorimetric assay was developed to detect myoglobin in biological samples [86]. The MIP was obtained from fluorescein *O*-acrylate and was used to capture myoglobin from test samples in a homogeneous assay. To ensure that the measured fluorescence signal was due exclusively to the myoglobin-bound MIP, superparamagnetic iron oxide nanoparticles (SPIONs) functionalized with myoglobin were added to the sample vial, after the MIP-sample binding reached saturation (5–10 min). The SPION particles were used to remove, by using a magnet, the excess, unbound MIP so that only the target protein-bound MIPs were left in suspension in the vial and the fluorescence signal was proportional to the quantity of myoglobin in the sample (Figure 6). Of note, the magnetic nanoparticles were obtained by a “green” synthesis method. The logarithm of the fluorescence intensity varied linearly with the logarithm of protein concentration in the range of 60 pg/mL to 6 mg/mL. The imprinting factor of the MIP is 1.95 and the non-specific binding, evaluated with BSA, amounted to 42.4%, about half of the response for the same concentration of myoglobin (3 mg/mL). At the same time, when analyzing spiked fetal calf serum samples spiked with myoglobin, the average recovery was 93%, indicating that the technique has adequate accuracy for myoglobin detection in biological samples. Together, these data show good potential for analyzing clinical samples but also the necessity to include control tests with non-imprinted polymer (NIP) particles to account for the non-specific effects. A wider interference study is necessary to prove the selectivity of the assay. By improving the imprinting factor and preventing non-specific adsorption effects, the performance of the assay can be enhanced further.

An alternative to starting from a fluorophore-labeled monomer for obtaining the MIP is to label the MIP with fluorescence-producing probes, e.g., with quantum dots (QD, [92]). An MIP-imprinted hydroxyethylcellulose membrane where the MIP was tagged with CdTe QD was used for the detection of myoglobin starting from 7.39 pg/mL (the lower limit of the linear range). The imprinted strip had a storage stability of (at least) 15 days. The detection was achieved in human serum that was diluted 1000 times with buffer. The interference study showed that cardiac troponin T, creatinine, and human serum albumin (HSA) do not significantly affect the sensor’s response when tested at concentrations ten times higher than the level of myoglobin. Nonetheless, some proteins, e.g., HSA, are present in much higher excess compared to myoglobin. Testing a “standard” panel of potentially interfering compounds at ratios reflecting those typical for clinical samples would be a big step forward to enable an objective evaluation of this and all other various sensing concepts proposed.

The challenges associated with measurements in complex samples such as serum are not trivial, as proven in another report on the detection of myoglobin [93]. The test relies on the conjugation of a dabcyl-labeled aptamer with a FAM-labeled partially complementary DNA sequence, resulting in the quenching of the FAM fluorescence signal in the absence of myoglobin and its specific recovery in the presence of myoglobin. The authors noted that proteins that are present in serum in high concentration, such as HSA, present at 35–50 mg/mL, induce a high fluorescence background. The fluorescence of proteins, attributed to the phenylalanine, tyrosine and—in particular—tryptophan residues in their structure is excited at 240–280 nm, and the maximum emission is in the range 300–350 nm. HSA contains one tryptophan residue. Nonetheless, at the very high levels found in serum, there is a strong overlap between protein’s fluorescence and that of the fluorophore label of the biosensor, even if in the above work the emission maximum of fluorescein is at 517 nm. Therefore, for accurate measurements of cTnI, the sample pretreatment was deemed necessary, i.e., diluting the serum by a factor of 10, and further purification by filtration through a 30 kDa cutoff centrifuge filter.

**Figure 7:**
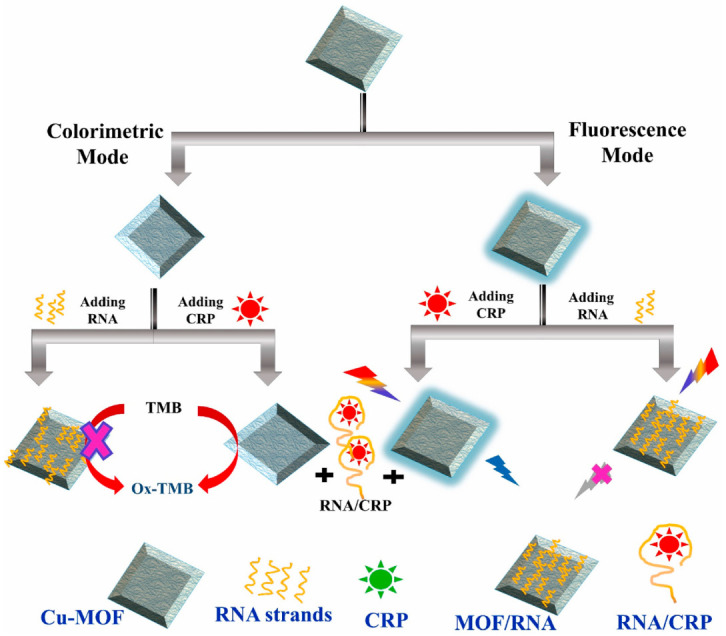
Principle of the detection of CRP by the dual colorimetry-fluorescence method relying on Cu-MOF and RNA-aptamer. Details are given in the text. Reproduced from [84] with permission from Elsevier.

In the recent period, the trends in developing multiplexed assays and assays based on dual detection modes continued. With regard to the latter, an illustrative example is a biosensor for the measurement of CRP by both colorimetry and fluorescence [84]. At the core of the assay stands a Cu-MOF material coated with an RNA aptamer specific for CRP. The Cu-MOF has peroxidase-like activity, functioning as a catalyst for the classic reaction of TMB and H_2_O_2_, resulting in the blue-colored compound measurable by colorimetry. The Cu-MOF also presents “stimulated fluorescence”, i.e., is not a fluorescent material per se but it is converted into one, following its reaction with H_2_O_2_; then, if excited at 320 nm, it will emit at 410 nm. The catalytic activity and the stimulated fluorescence properties of the Cu-MOF are both inhibited when the material is coated with the aptamer and recovered when the aptamer is desorbed following its interaction with the CRP in the sample. The approach enables the obtainment of two reliable results (by fluorescence and colorimetry) with one platform (Figure 7). The size of Cu-MOF particles is in the sub-micrometer-to-micrometer range, as characterized by Field Emission Scanning Electron Microscopy (FESEM). A selectivity study was conducted for both detection techniques and concluded that the signal for CRP was high and selective when compared to several compounds tested with 100 times more concentrated solutions than CRP. Nonetheless, in real serum fluid, the quantity of some biomolecules such as serum albumin is more elevated than the tested amounts. Further investigations are thus necessary, and they need to include other cardiac biomarkers as potential interferents in order to definitively prove that the analytical system is adequate as a diagnosis tool. When the method was applied to spiked diluted serum, the recovery percentages were 84–102%, showing good accuracy for real sample testing. Unfortunately, the analysis of a set of clinical samples and the comparison with a standard method were not addressed and remain the next steps for advancing the proposed concept.

The multiplexed detection of eight biomarkers with a single strip was demonstrated by Huang et al. [91], who combined the analysis of Myo, CK-MB, and cTnI by a fluorescence sandwich immunoassay with the determination of cholesterol (TC), triglyceride (TG), high-density lipoprotein cholesterol (HDL-C) and uric acid by dry chemistry. Additionally, the content of low-density lipoprotein cholesterol (LDL-C) in the sample was derived by calculation. The strip was intended as a diagnostic tool for acute myocardial infarction (AMI). Towards this goal, the selectivity for cTnI detection was first proven by comparing the signals for cTnI (50 ng mL^−1)^ with those for CK-MB (500 ng mL^−1^), Myo (500 ng mL^−1^), TC (50 mmol L^−1^), TG (50 mmol L^−1^), UA (50 mmol L^−1^), and HDL-C (50 mmol L^−1^). The analytical performances of the strip feature, among others, detection limits for Myo, CK-MB and cTnI of 10 pg/mL, 2 pg/mL and 1 pg/mL, respectively. Following detailed characterization, the sensor strip was used to analyze a set of serum samples collected from AMI patients, and the results were compared with the current clinical methods based on chemiluminescence immunoassay (CLIA). The good correlation between the two sets of results stands as evidence of the applicability and usefulness of the proposed 8-in-1 test.

**Figure 8:**
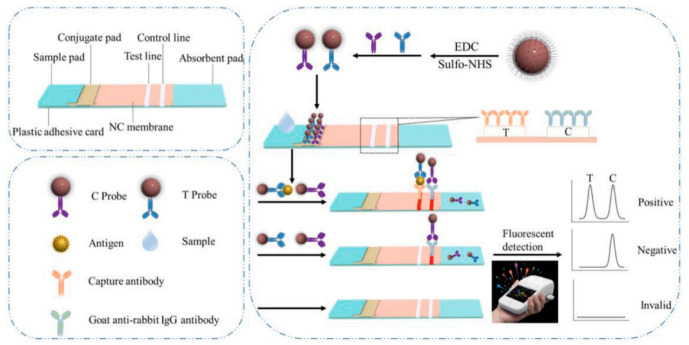
Principle of the QBs@SiO2-COOH-based LFIA and of the detection of cTnI. Details are given in the text. Reproduced from [81] with permission from Elsevier.

In the search to enhance the sensitivity of the measurements and reduce the time per assay, new materials are critical. CdSe/ZnS quantum dots of 14 nm medium size were assembled into nanobeads by encapsulation in cetyltrimethylammonium bromide (CTAB) and were subsequently coated with SiO_2_ and polyvinylpyrrolidone to preserve their luminescence properties in various environmental conditions [81]. The coated beads (QBs@SiO2-COOH) with an average diameter of 235 nm displayed an enhancement of 1967 times in luminescence and a remarkable stability in complex samples and at different pH and temperatures [81]. The principle of the test is classic for an LFIA, where the ratio between the fluorescence signal at the test and control lines are proportional to the biomarker concentration in the sample (Figure 8).

Nonetheless, the strong luminescence and optimized ratio between the nanobeads and the cTnI antibodies were the key to achieving a detection limit of 0.036 ng mL^−1^, i.e., about 20 times lower than the smallest concentration detectable by a similar LFIA using SiO_2_-coated QDs. The stability of the sensor strips was evaluated based on the measurements for four concentrations of cTnI, and the results support the stability of the strip stored at either room temperature, 37 °C or 45 °C for 120 days. By appropriate modification with specific antibodies, the sensor was used for the simultaneous measurement of CK MB, Myo and cTnI. Remarkably, a set of 38 human serum samples were analyzed in parallel with the proposed QBs@SiO2-COOH-based LFIA and by ELISA, and it was determined that the concentrations of cTnI, CK-MB and Myo measured by the two methods were linearly correlated, with the slopes of the fitting lines being very close to 1 and correlation coefficients R = 0.980–0.991. The assay time with this strip was 10 min and the reproducibility of the test was adequate, i.e., the CV of intraassay and inter-assay tested at three concentration levels of cTnI were below 8%. Of note, a large set of 103 clinical serum samples were analyzed for their level of cTnI in parallel by the LFIA and by a standard chemiluminescence method, and the results were in good agreement, supporting the accuracy of the LFIA. This work is a very nice illustration of detailed, very informative research reporting on all of the stability characteristics, analytical performances, feasibility for testing clinical samples and comparison with standard methods. The benefits of the new nanobeads material are clear as they are quantitatively evaluated in comparison with the starting, simple QDs (in terms of luminescence intensity and detection limit achieved for cTnI). This work serves as a model for studies aiming to close the gap between the research laboratory and clinical practice.

In summary, fluorescence enables high flexibility in designing the detection approach due to the variety of fluorochrome probes and dyes with unique properties. Fast, simplified POC detection enabled by portable readers combined with LFA appears to be the main avenue of research and vector of progress towards commercially available devices. Some of the recent studies focus on new materials and specific receptors for achieving a large linear range and great sensitivity. The search for ultrasensitivity of detection was prompted by the desire to depart from the traditional approaches based on invasive blood testing towards more patient-friendly procedures, e.g., testing of saliva [82] or analysis from very low volumes of blood. Thus, efficient signal amplification was ensured, e.g., by deoxyribonuclease I-aided target recycling [82], and in some of these works, issues such as selectivity, real sample testing and verifying the accuracy with standard assays remain to be addressed. A glimpse at the data in Table 4 emphasizes that in many cases the verification of accuracy was limited to spiking and recovery studies. Not all concepts were verified with clinical samples and compared to standard or current methods in clinical laboratories. While the main reason for switching from antibodies to MIP is to gain stability, this aspect was rarely investigated in detail for long time periods. Despite the progress in the biomarker analysis, there are significant challenges related to the complex composition of the serum samples that new assays relying on new materials and recognition mechanisms must overcome. Works reporting validation tests and the analysis of large sets of clinical samples, multiplexed detection for analyzing specific CVDs and combining different detection modes in the same analytical platform converge with studies on new materials and hint at a promising future of fluorescence-based tests for CVD biomarkers.

### 3.4. Chemiluminescence- and Electrochemiluminescnce-Based Biosensors

Chemiluminescence (emission of light induced by a chemical reaction) and electrochemiluminescence (ECL, where the luminescence is triggered by chemical species formed in an electrochemical reaction) facilitate the extremely sensitive detection of various analytes, including CVD biomarkers (Table 5).

For example, a chemiluminescence assay for cTnI requiring 5 µL of serum sample relied on a microfluidic chip and magnetic beads modified with an aptamer for the specific capture of the protein biomarker [94]. The captured cTnI was bound to a primary antibody that was further linked to an HRP-labeled anti-IgG antibody. The signal was due to a chemiluminescent HRP substrate and varied linearly with the cTnI concentration in serum in the range of 0.196 to 3.931 ng/mL. The accuracy of the assay was proven by recoveries of 90–108.5% from spiked serum samples and by the similarity of results (i.e., within 1.14–7.57%) with standard chemiluminescence immunoassay performed with the commercial Siemens ADVIA Centaur systems for five human serum samples containing between 16.3 and 927.3 ng/L cTnI. Remarkably, the microfluidic chip-based platform enabled the analysis of six samples in 30 min.

Multiplexed, shorter analysis of cTnI, CK-MB, and Myo (within 17 min) was achieved with another microfluidic platform with chemiluminescence detection [95]. In a classical manner, the specificity of the detection was ensured via an antibody-based sandwich while the chemiluminescence was produced in a reaction catalyzed by HRP, used as a label for the detection antibodies. Nonetheless, mixing the detection antibodies with the sample upon introducing it into the microfluidic chip enabled the shortening of the assay time compared to ELISA. Obtaining the chip by 3D printing was reported to reduce the costs.

An ECL assay was developed by [96] for the detection of NT-proBNP down to a detection limit of 0.11 pg/mL and a linear range between 0.25 pg/mL and 100 ng/mL. The signal generation was based on the electrochemiluminescence-resonance energy transfer between gold nanoparticles modified with silver nanocubes (AgNCs) and a metal–organic framework of type MIL-125. The strategy for obtaining the donor luminophore by coating AgNCs with semicarbazide and attaching them to Au nanoparticles resulted in a stable ECL signal with a three times higher intensity compared to that provided by AgNCs alone. The modified particles were deposited on an electrode, and a primary antibody specific to NT-proBNP was attached to the donor luminophore. The Ti(IV)-based metallic organic framework MIL-125 has an adsorption spectrum overlapping with the emission spectrum of the donor and quenched the luminescence of the donor. Thus, when NT-proBNP was sandwiched between primary and secondary antibodies fixed on the donor and acceptor particles, respectively, the decrease in ECL was directly correlated with the concentration of the cardiac biomarker in the sample. The approach led to similar results to the classic ELISA, and the accuracy was proved by the good recovery (96.8–100.2%) in experiments with human serum spiked at three concentration levels.

While used in standard clinical laboratory equipment, these detection methods were less represented in the biosensors field compared to, e.g., fluorescence, unravelling great sensor development opportunities for the near future.

### 3.5. Colorimetry-Based Biosensors

Colorimetry is a simple optical method that measures the color change when modifications occur as a result of a reaction in solution [97] or on a surface (e.g., lateral-flow assay). Colorimetry is very promising in developing POC tests because it can be rapid and cheap and can be carried out by unskilled personnel.

Consequently, several studies in recent years used this detection method for the analysis of CVD biomarkers. Data summarized in Table 6 show a variety of colorimetry-based approaches aimed at sensitive and accurate detection, meeting the clinical cutoff for specific CVD biomarkers in serum samples.

Paper-based, microfluidic chips or solution-based assays were reported (Table 2). Most analytical approaches are derived from sandwich-type immunoassays by replacing enzymes with nanozymes [84,105,109] and DNAzymes [108]. This corresponds to a growing trend compared to the previous period, as is the case also with the development of aptamer-based assays, which increased in the context of continuing efforts to select new specific sequences with high affinity for CVD biomarkers (e.g., for cTnT, [107]. Oligonucleotides enable additional sensing strategies compared to antibodies including new approaches for signal generation and amplification, e.g., supersandwiches made by DNA hybridization [97], DNAzymes [108] and Exo -I assisted amplification [109]. Increased stability is the major goal driving these changes from enzymes and antibodies to nanozymes/DNAzymes and aptamers, respectively. From this perspective, the lack of stability data on these new materials and sensors is intriguing and disappointing.

The time per assay ranged from 1.5 to 20 min [98,102,103,104], indicating potential for POC testing, to more than several hours (Table 2). Undiluted serum was used in several LFIA [104] and homogeneous assays; however, in general, dilution with buffer was found to be an adequate procedure to bring the sample concentration in the linear range of the method and minimize interference.

**Figure 9:**
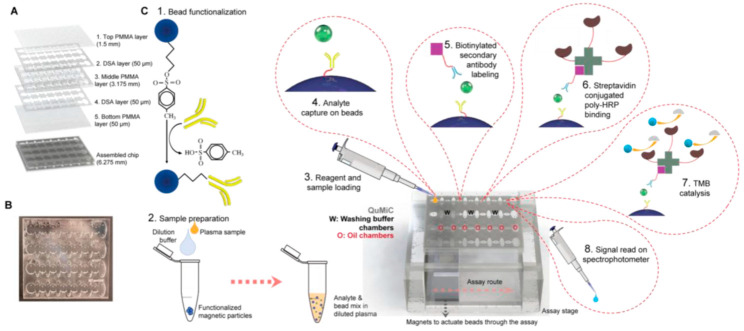
Construction and working principle of the Total Microfluidic chip for Multiplexed diagnostics (ToMMx). (**A**) Polymethyl methacrylate (PMMA) and double-sided adhesive (DSA) polyethylene terephthalate (PET) film layers of ToMMx design. (**B**) Laser-cut and assembled ToMMx. (**C**) Bead functionalization, sample preparation and assay steps of ToMMx. (**1**) Functionalization of tosyl-activated magnetic beads with analyte-specific primary antibodies. (**2**) Sample dilution buffer, plasma sample and functionalized beads mixed in tube as sample preparation. (**3**) Assay reagents, buffers and sample loading on ToMMx. (**4**) Analyte in the sample captured on antibody-functionalized beads. (**5**) Analyte–antibody complex labeled with biotinylated secondary antibody. (**6**) Streptavidin conjugated poly-HRP binding to antibody–antigen–antibody sandwich complex. (**7**) TMB substrate catalysis by poly-HRP in the complex. (**8**) Evaluation of analyte concentration via color change in the sample after transferring the colored liquid to a 96-well plate, mixing with stop solution and reading in with a spectrophotometer. Reproduced from [102] with permission from Elsevier).

Screening several CVD biomarkers simultaneously is time-saving and helps to establish the type of CVD. Ozen et al. [102]. developed a Total Microfluidics platform for multiplexed diagnostics (ToMMx) for the detection of cardiac troponin-I (cTnI), heart-type fatty acid binding protein (hFABP) and N-terminal pro-brain natriuretic peptide (NT-proBNP) (Figure 9). The assay included similar steps to ELISA, with modifications adapted for ToMMx. The platform is filled iteratively with mineral oil, washing buffer and water-based reagents with the help of surface tension differences, so these components did not mix. They were preloaded before sample processing. The magnetic beads employed in the assay are actuated using a magnet. The analytes are specifically captured between Ab immobilized on the beads and a biotinylated secondary Ab, then a complex is formed when adding streptavidin-conjugated poly-HRP. The analytes’ concentration is evaluated by color changes when TMB added as a substrate whose oxidation is catalyzed by HRP. A set of clinical samples was analyzed in parallel by the platform and by standard ELISA, and the results showed that the proposed method is precise and accurate. The sample set included 38 patient samples corresponding to different types of CVDs and 12 control samples. Using the platform’s results, the patients were diagnosed with an accuracy of 91% for acute coronary syndrome (via cTnI and hFABP) and 95% for severe symptomatic aortic stenosis (via NT-proBNP), respectively. When NT-proBNP was used as a diagnostic biomarker, its detection with this analytical platform led to the identification of patients suffering from dilated cardiomyopathy with 100% accuracy.

Even if these results are very promising, a wide selectivity study is necessary to validate the method. The total time of the assay is less than 20 min, which is 15-fold reduced compared with ELISA. In the future, the authors predicted that by integrating the ToMMx platform with portable detection systems such as smartphones and by mass production of the assay kit, this multiplex method can become easy and cheap, accessible for everyone.

With the same aim of simplified assays for CVD biomarker detection, Wang et al. [108] developed an ingenious instrument-free detection method for cTnI, where the length of a “coffee ring”-type colored band, developed on a microfluidic paper device—simply measurable with a ruler—was proportional with the concentration of the cTnI in the sample. The making of this microfluidic device involved a pair of antibodies (for the sandwich-type sensing) as well as oligonucleotides (to form a DNAzyme) and other reagents: hemin (for the DNAzyme), iodide and H_2_O_2_ (DNAzyme substrates) and starch (for the blue color development by reaction with iodine). The work nicely illustrates the versatility of DNA hybridization, which in this case served to assemble the DNAzyme. Specifically, the detection antibodies were labeled with oligonucleotides that hybridized to complementary DNA to form a G quadruplex. In the presence of hemin, this acts as a peroxidase mimic catalyzing the oxidation of iodide to iodine.

In another report, DNA hybridization was used to (i) attach the aptamer to a capture probe fixed on magnetic beads, (ii) assemble the enzyme-labeled signaling probe [97] and (iii) bind the signaling probe to the aptamer. The quantitative test for myoglobin was performed in a test tube, and an optical amplifier was used as a readout instead of a spectrophotometer to simplify the equipment needed for the quantitative detection. Developed as a turnoff assay, the measurement relied on the proportionality between the decreased amount of HRP-labeled probe hybridized to the aptamer (and consequently, lower color intensity following the enzymatic reaction) and the quantity of myoglobin in the sample, bound by the aptamer. This test required almost 3 h from which the longest step (90 min) was the attachment of the DNA-based, HRP-labeled “supersandwich” signaling probe. Consequently, the time per assay remains an aspect to be significantly improved in the future.

New aptamers were recently selected for cTnT [107], targeting different epitopes of the biomarker and with affinities of K_D_ = 122 ± 14 nM (Apt1) and 190 ± 20 nM (Apt2). These were used to develop an enzyme-linked oligonucleotide assay (ELONA). The work emphasized one important advantage of the sandwich over the direct detection format; namely, it enabled the mitigation of matrix effects and obtained the same sensitivity when analyzing cTnI in undiluted serum as in the buffer solution.

Aiming to replace enzymatic labels to increase the stability and reduce costs, while allowing for multiplexed colorimetric sensing, Pu et al. [99] reported the use of phenolphthalein, methyl red, and bromothymol blue dyes as labels enabling the specific, simultaneous immunoassay of three biomarkers for AMI (NT-proBNP, CK-MB and cTnT). The dyes with a pH-dependent color were loaded on Au nanovesicles and their loading/release was temperature-controlled. The nanovesicles were functionalized with specific antibodies and the assay was performed similarly to a classic ELISA. Comparative analysis of a set of serum samples via the proposed test and by a standard immunofluorescence assay typically used in a clinical laboratory indicated similar results. The new assay has the benefits of higher sensitivity and a wider linear range.

Xie et al. [106] described an antibody-free, ELISA-like assay for CRP in which the biomarker was sandwiched between a conjugate of citicoline and BSA as a capture probe and an aptamer as a detection probe. The aptamer was labeled with AuNPs acting as an HRP-mimicking nanozyme while TMB was used as the nanozyme’s substrate. Remarkably, the reproducibility of the materials used in the test, i.e., the citicoline-BSA conjugate and the AuNPs labeled aptamer, was assessed by performing the analysis of a serum sample with five lots. Low, acceptable variations between batches of materials were found as indicated by the CV of 7.11% for the CRP concentration in the sample. The accuracy of the test was demonstrated by the similarity of results obtained by the proposed assay and classic ELISA, performed in parallel.

While noble metals are increasingly used as colorimetric probes and as nanozyme labels, kit developers have struggled to minimize the amounts of these expensive and rare materials [105]. For example, Panferov et al. [105] integrated trimetallic nanoparticles made from Au, Ag and Pt into a lateral flow immunoassay (LFIA) for C-reactive protein (CRP) where Pt atoms were dispersed on the surface of nanoparticles rather than forming a full coating. With this nanozyme, the detection limit was improved to 15 pg/mL CRP, i.e., 65 times compared to using AuNPs alone, while the ratio material/catalytic performance was minimized. It is also important to mention the same authors’ preoccupation with less invasive approaches as, besides testing serum samples, the researchers performed some preliminary studies with capillary blood. While acknowledging that the background interference found for some samples necessitates sample pre-treatment, the problem might find an easy solution in the future, and the use of capillary blood appears to be an avenue worth investigating. In view of the adoption as a POC device, the assay will benefit from further simplification to eliminate the need for the enzymatic substrate, 3,3, diaminobenzidine and the H_2_O_2_ to be added separately. Nonetheless, the measurements with the described LFIA were performed in less than 10 min.

A very recent report combining aptasensing with nanozymes for the detection of cTnI [109] included several innovative aspects. For increased efficiency, the nanozyme-catalyzed reaction was confined in pores of tungsten trioxide (p-WO3) serving as reactors. The color-producing signal readout was based on “enzymatic” inhibition and a less usual metallic organic framework nanozyme was employed, MOF-818 with catechol oxidase-like activity. The performance of the nanozyme towards the oxidation of 3,5-Di-tert-butylcatechol (3,5-DTBC) drastically improved upon confinement into the 400 nm pores of WO_3_, showing a Michaelis Menten constant of 1.42 mM, a catalytic yield of 95.2% and a rate constant of 31.47 s^−1^ compared to 2.49 mM, 26% and 9.94 s^−1^, respectively, in solution. Moreover, the test integrated a signal amplification step assisted by Exo-I, as another illustration of the variety of configurations and sensing approaches enabled by the use of oligonucleotides.

**Figure 10:**
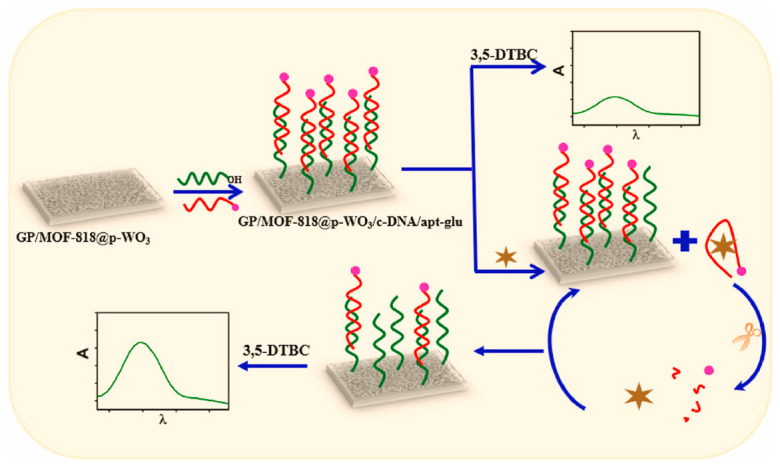
The assembly and working principle of the GP/MOF-818@p-WO3/c-DNA/apt-glu aptasensor for the analysis of cTnI. The glutathione label, cTnI and Exo I are depicted by a pink ball, star and scissor like symbols, respectively. The absorbance (A) at λ = 425 nm of a solution of 3,5-DTBC incubated with the sensor is quantitatively correlated with the amount of cTnI in the sample. Details are given in the text. Reproduced from [109] with permission from Elsevier.

In more detail, a glass plate was coated with the nanozyme/p-WO_3_ material and a DNA sequence, complementary to the cTnI aptamer, was covalently bound to this material (Figure 10). Next, the cTnI aptamer, labeled with glutathione, was anchored to the surface by hybridization to the cDNA. In the presence of 3,5-di-tert-butylcatechol (3,5-DTBC), the assembled sensor produced low absorbance at 425 nm as the substrate’s conversion was limited due to the presence of glutathione, a known inhibitor of catechol oxidase. The sensor was then incubated with the sample containing cTnI and Exo1. The aptamer, having a high affinity towards cTnI, desorbed from the sensor surface while the Exo I in solution cut the aptamer and enabled the recovery of cTnI, which bound further to the surface, repeating the cycle for an amplified desorption of the aptamer. In the last step, the sensor was incubated again with the substrate, and since the inhibition due to glutathione was removed following the desorption of the aptamer, the catalytic activity of the nanozyme was recovered. The conversion of the substrate, expressed through the increase in the absorbance at 425 nm, was proportional to the amount of desorbed aptamer and by consequence to the cTnI in the sample. This sensing configuration enabled the achievement of a detection limit of 18 pg/mL cTnI [109]. While the method presumes three steps totaling more than 30 min, it was shown that it can be applied to undiluted serum samples with good accuracy (i.e., 95–107% recovery for four spiked samples; the analysis of the unspiked healthy serum was in agreement with the results of a parallel ELISA test). Moreover, the optical aptasensor can be reused multiple times, the decrease in response after 30 uses being lower than 15% (estimated based on the data presented in [109].

To summarize, the colorimetry-based approaches discussed above for the detection of CVD biomarkers reflect sustained research efforts towards (i) improving the detection sensitivity using new nanomaterials or image processing methods, (ii) simplifying the equipment (e.g., using a smartphone as a read-out tool or measuring the width of the colored bands using a ruler, using an optical fiber amplifier), (iii) improving the stability (e.g., by replacing enzymes with nanozymes and DNAzymes), (iv) developing multiplexed platforms for the detection of multiple biomarkers relevant for the diagnosis of specific CVDs and (v) developing dual or multimodal detection strategies. New detection mechanisms (e.g., using enzymatic inhibition), new labeled probes (e.g., glutathione labeled aptamer) and new aptamers were proposed. At the same time, disappointingly, there was no evaluation of the stability and reproducibility of the new materials, with very few exceptions. Some lateral flow strips were evaluated and found to be stable for months at either 4 °C [110] or at room temperature [104].

The progress in the field of nanomaterials has led to increasingly more applications in sensing. In particular, major gains were related to nanomaterials’ role as catalysts (i.e., nanozymes; Refs. [106,109], in comparison with the more “traditional”, use as high-loading capacity carriers for colorimetric probes or recognition molecules [99] or as signaling probes themselves [101,104].

The vast majority of reports describe the analysis of clinical samples and their comparison with current procedures implemented in clinical laboratories (Table 2). Some works were limited to the analysis of a few samples of spiked serum, indicative of the applicative potential and adequate accuracy of the new methods for CVD diagnosis. Nonetheless, many of the studies went further to include the application of the new methods for testing between a few and fifty patients [102]. Particularly remarkable are reports on multiplexed detection of several biomarkers targeting the diagnosis of specific CVD (see the review of [9], which includes data on their diagnostic accuracy [102]). This trend is encouraging for the future development of the proposed methods into commercial kits and devices.

### 3.6. Other Optical Methods

Other optical methods used more rarely with biosensors for the detection of CVD biomarkers include retroreflection, dynamic light scattering and microfiber Bragg grating (Table 7).

Retroreflection is an optical phenomenon that consists in the reflection of the incident light back to the light source by a specific surface called a “retroreflector”. This optical phenomenon, widely exploited to make traffic signs and reflective tape for clothing, was considered recently for signal transduction in the field of biosensors [111,114,115]. The use of polychromatic instead of monochromatic light reduces the cost of optical detection, while the compatibility with the smartphone LED flash and camera is an additional huge asset of retroreflection devices. Thus, it enables the simplification of the required equipment, e.g., by integrating both the light generation and the detection parts in a smartphone. Microsized retroreflective surfaces that can function as optical signal labels in biosensors were devised from Janus particles, e.g., silica spheres with one hemisphere coated with a retroreflective layer, made by successive deposition of aluminum and gold layers [111,114,115]. A 2022 report by Kim et al. [111] describes a microfluidic chip used with retroreflective particles for the detection of CK-MB in spiked serum by an approach that circumvents the washing steps. The capture antibody is anchored on the transparent microfluidic chip. The detection antibody is specifically attached to the silica half-sphere of Janus particles, as the gold-coated hemisphere of the Janus particles was first blocked with 6-mercaptohexanol. The biomarker CK-MB becomes “sandwiched” between the capture and the detection antibody. By flipping the microfluidic chip, the unbound Janus particles are sedimented away from the chip’s surface. This ingenious, wash-free approach enabled the user-friendly detection of CK-MB in 1 h using a device integrating a smartphone.

The formation of particle aggregates facilitated by the binding of a target biomarker stands as the basis of some alternative approaches for the detection of CVD biomarkers. The aggregates’ formation influences the light scattering properties and turbidity of the solution. Dynamic light scattering (DLS) exploits the Brownian motion of particles in a solution and measures the variation in time of the intensity of the light scattered by these particles, the scattering being observed at a fixed angle, typically 90° compared to the incident light. Particles with a size within a range from nanometers to a few micrometers are measured in a non-destructive way, very fast, in less than a minute. Based on the principle of aggregate formation, the detection of NT-proBNP was achieved in under 20 min, down to 7.4 fg/mL by DLS [112]. The test relies on the use of (i) magnetic beads modified with antibodies specific for NT-proBNP and (ii) silica particles modified with 3-aminophenylboronic acid. Aggregates of the two kinds of particles are formed in the presence of NT-proBNP, which binds both to the magnetic beads (via the antibody) and to the silica nanoparticles (by boronate affinity). The hydrodynamic diameter of the aggregates varies linearly with the concentration of NT-proBNP in the range of 12 fg/mL to 100 ng/mL. The selectivity of the assay was first proven with a set of monosaccharides and glycoproteins, after which the test was applied for the analysis of a set of 40 clinical samples. Moreover, the results were compared with a standard time-resolved fluorescence immunoassay (TRFIA) used in the clinical laboratories. The sensitivity for NT-proBNP was better than that of the standard assay, and the results were linearly correlated to those provided by the standard method (correlation coefficient: 0.9745), indicating that the proposed assay is accurate and adequate for clinical samples. Notably, the assay requires a very low sample volume (1 µL).

Microfiber Bragg grating (mFBG) probes have a good potential to be used for in vivo and at-patient monitoring of biomarkers due to their sensitivity, compactness and multiplexing possibilities [113]. An FBG is an optical fiber in which the refraction index changes in the longitudinal axis, enabling, in essence, to modulate what wavelengths will be reflected and what will be transmitted by the fiber. Towards advancing from the theory to practice, Ran et al. developed a harmonic optical mFBG immunosensor for detection for cTnI [113] based on a fiber functionalized with a specific antibody. The difference in the reflection spectrum acquired at the second and third harmonic resonances enabled us to distinguish between the temperature effect and the specific sensor signal. Owing to the faster binding kinetics at higher temperatures, the detection of cTnI in serum can be significantly shortened, from 1 h to 25 min if the measurements are performed at 37 °C rather than at 25 °C.

## 4. Challenges in the Development of Optical Biosensors for CVD

The major hurdles in the development of optical biosensors for CVD are related to proving the performances of the new analytical tools by analyzing large sets of clinical samples and comparing them with standard methods in clinical laboratories. This is the critical step for launching the proposed devices and methods towards commercial applications and widespread adoption. However, until then, there are some smaller but very important issues to address, such as the assessment of the specificity and accuracy of the proposed methods.

A brief look at the specificity studies summarized in Table 2, Table 3, Table 4, Table 5, Table 6 and Table 7 shows the large variations between studies with respect to the number of potentially interfering molecules that were evaluated. Moreover, their quantitative ratio compared to the target analyte was also very variable among the studies and did not always reflect the actual ratios expected in the biological samples. Concerning proving the accuracy, this was often evaluated exclusively based on spiked serum samples, and the calculated recovery was excellent, with several concentration levels often evaluated in the same study. This is an important achievement; nonetheless, analyzing a set of different samples large enough to reflect differences in the sample composition, not only with respect to the target analyte but also to the matrix itself, would be more convincing. A practical problem that is evidenced when looking closer at the spiking protocols is the incorrect manner still used by some researchers, where the sample (serum) was first diluted and then spiked, instead of spiking first and then performing any sample pre-treatment including dilution. These examples highlight the need for adhering to uniform, adequate experimental protocols for evaluating the specificity and accuracy of the proposed biosensors and assays.

Reconciling the analysis of several biomarkers with a small sample size is another challenge that could be addressed by novel, high-sensitivity biosensors and assays.

Salivary biomarkers are increasingly researched in the quest for establishing less invasive analysis procedures [116]. Nonetheless, so far, the number of studies focused on testing CVD biomarkers in saliva is small, the number of samples analyzed is also low and those analyzed in parallel to blood samples are even fewer. Consequently, there are no clearly established correlations with blood levels of the same biomarkers. The analysis of saliva remains difficult due to the high sensitivity of the methods required to accurately analyze the samples (levels being even 1000 times lower than in blood) and also due to several factors of variation. So far, there have been some indications that natriuretic peptides are useful indicators of heart failure. Recent studies validated the use of reference methods such as ELISA and established the stability of saliva samples. As noted by Rammos et al. [116], a panel of several biomarkers, rather than the analysis of individual compounds, will provide a good correlation with standard methods that use blood as a sampling matrix and thus will promote the acceptance of saliva testing in the medical community. Nonetheless, more and larger studies need to be conducted to obtain statistically relevant data.

When developing an optical biosensor for the determination of CVD biomarkers, the transduction methods discussed above enable very sensitive detection and have distinct advantages and limitations (Table 8).

The measurements by all these methods are very fast, the time per assay with optical biosensors mainly being dictated by the duration of the binding, washing (if necessary) and amplification steps. Moreover, looking at the data summarized in Table 2, Table 3, Table 4, Table 5, Table 6 and Table 7, it is also obvious that both the label-free methods and the label-based methods are useful for developing high-performing biosensors for the detection of CVD biomarkers. The simplicity of some label-free methods comes at the cost of a compromise in performance, while tests involving the use of optical labels might involve higher costs. Additionally, choosing a sandwich-type detection format improves the detection limit by more than an order of magnitude compared to direct detection, e.g., for cTnI, the LOD of an SPR biosensor reached 0.028 ng/mL and 0.43 ng/mL for sandwich and direct assay, respectively [43]. However, the time per assay and costs increase as well. Consequently, while the information in Table 2, Table 3, Table 4, Table 5, Table 6, Table 7 and Table 8 may serve as a guide to a developer of optical biosensors for CVD biomarkers, the main recommendation is to choose the most suitable approach for the envisaged application. For example, if real-time monitoring of binding for screening a series of ligands or drugs or if the calculation of kinetic constants of new ligands is targeted, SPR is a very good choice. If the non-destructive analysis of the sample is important, e.g., to enable subsequent testing by another method, SERS would be preferable. Furthermore, if fast, cost-effective testing is a priority, then colorimetry will serve as a starting point for developing new analytical devices etc.

## 5. Conclusions and Perspectives

The field of optical biosensors has progressed at a fast pace with the aim of developing ever-more specific and sensitive, faster and more convenient devices, i.e., simpler and cheaper. Multiplexed detection was increasingly researched for facilitating faster diagnosis and monitoring of specific CVDs.

Several solutions were advanced to shorten the time per assay, including, e.g., by conducting the measurements at higher temperatures for faster binding kinetics. Progress was also made with respect to improving the portability of the equipment, for simplification and cost reduction.

Other advancements were directed at making the tests less invasive to encourage patient compliance. The strategies pursued varied from reducing the sample volume per test to a few µL (similar to glucose tests) to using alternative sample matrices such as saliva.

The current wealth of knowledge also enabled setting new ambitious goals for the future:

- new biomarkers or combinations of biomarkers will be proposed as relevant for CVD; their discovery and determination will involve a huge amount of data whose interpretation can be facilitated by artificial intelligence (machine learning) approaches
- new biorecognition receptors await discovery; in particular, it can be anticipated that more stable aptamers, MIPs together with nanobodies, will be screened for their specificity towards established or new biomarkers. With regards to aptamers, DNA amplification and editing techniques will likely see increased applications in the optical sensing of CVD biomarkers.
- continuous development of optical readers and disposable tests with fast reading will enable the lowering of prices and simplification to the point of facilitating at-home testing—similar to glucose testing for diabetic persons.

## Figures and Tables

**Table 1:** Examples of POC and currently used procedures for the measurement of CVD biomarkers in hospitals and healthcare units.

Name of the Test	Method Details	Analyte	LOD (ng/mL)	Time per Assay	Sample Size	Reference
Human ELISA kit, Thermo Fisher Scientific Inc., Waltham, MA, USA	Colorimetric Microplate Reader	cTnI	1 × 10^−1^	4 h 45 min	50 µL	[14]
Abbott i-STAT 1™ Troponin I, Abbott Point of Care Diagnostics, Princeton, NJ, USA	Electrochemical (amperometric) sensor	cTnI	2 × 10^−2^	10 min	14 µL	[15]
Roche cobas h 232 POC system Roche Cardiac test strips (Roche Diagnostics, Basel, Switzerland	LFIA	cTnT NR-proBNP CK-MB D-dimer Myo	Range: cTnT: 4 × 10^−2^–2.0; NT-proBNP: 6 × 10^−2^–9.0 CK-MB: 1.0–40 D-dimer: 1 × 10^2^–4 × 10^3^ Myo: 3 × 10^1^–7 × 10^2^	12 min (cTnT, NT-proBNP, CK-MB); 8 min (D-dimer, Myo)	150 µL	[16]
Elecsys NT proBNPII on Cobas (Roche Diagnostics, Basel, Switzerland)	ECLIA	NT-proBNP	5 × 10^−3^	18 min	9 µL	[17]
CK-MB/cTnI/Myo Fast Test Kit (Immuno-fluorescence Assay) (Getein Biotech Inc., Nanjing, China)	Fluorescence	CK-MB	CK-MB: 2.5	10 min		[18]
		cTnI	cTnI: 0.1			
		Myo	Myo: 30			
Advia CentaurXPT Immunoassay System, (Siemens, Munich, Germany)	Chemiluminescence	BNP, CKMB, cTnI, Myo NT-proBNP	cTnI: 1.24 × 10^−3^	18 min; high throughput: 240 samples/h	10–200 µL	[19]
Abbott NT-proBNP assay on the Architect i2000 analyser (Abbott Laboratories, Chicago, IL, USA)	Chemiluminescent microparticle immunoassay	NT-proBNP	4.9 × 10^−3^			[20]

**Table 2:** Performances of recent SPR-based methods for the detection of CVD biomarkers.

Biorecognition Element	Detection Details	Analyte	LOD (ng/mL)	LR (ng/mL)	Selectivity Study	Analysis of Real Samples	Reference
Antibody	SPR imaging	cTnI	1.2 × 10^−4^	10^−3^–8	Myo, IgG, PSA	Clinical samples (serum)	[41]
Zwitterionic peptide-based aptasensor	Angle-scanning SPR system; Kretschmann configuration	cTnI	20	20–6 × 10^2^	BSA, Lys, HSA	Spiked fetal bovine serum	[42]
Protein G, antibody	LRSPP waveguides	cTnI	4.3 × 10^−1^ (direct) 2.8 × 10^−2^ (sandwich)	1–10^3^	-	-	[43]
Antibody	LSPR Au nanoprisms	cTnT	5.25 × 10^−7^	1.75 × 10^−5^–3.5 × 10^−2^	Tropomyosin	Plasma, serum, urine	[44]
Antibody	Fiber-optic-based SPR	NT-proBNP	10^−2^	10^−2^–10^2^	-	-	[45]
Antibody	Amplified SPR with hollow gold nanoparticles and magnetic probes	cTnI	1.25	-	Mouse IgG, bovine IgG	Spiked human serum	[46]
Antibody and aptamer	Amplified SPR with gold nanoparticles and magnetoplasmonic nanoparticles	BNP	2.82 × 10^−5^	10^−4^–10^−1^	BHb, AA, Mb, OVA, BSA	Clinical samples (serum)	[47]
Antibody	PhotonicSys SPR H5, Bimetallic SPR chip	NT-proBNP and S100β	12 and 7.5 × 10^−1^	2.5 × 10^−1^–10	-	Plasma	[48]

**Table 3:** Performances of recent SERS-based methods for the detection of CVD biomarkers.

Method Details	Analyte	LOD (ng/mL)	LR (ng/mL)	Selectivity Study	Analysis of Real Samples (Number of Samples)	Reference
Aggregated Au NPs’ nanofluidic device	BNP cTnI CRP	-	-	-	-	[54]
Aggregated AuNP;Ab conjugated agarose beads	CRP	-	-	-	-	[55]
Combined SERS-ELISA; sandwich immunoassay; AuNPs; TMB^2+^	cTnT	2 × 10^−3^	2 × 10^−3^~3.20 × 10^−1^	-	Two human serum samples	[56]
Optofluidic device comprising plastic plates, rubber layers, a nanoporous membrane, rhodamine-6G labeled myoglobin and colloidal AuNPs.	Myo	-	-	Decrease in the SERS signal in the presence of BSA	-	[57]
Three-dimensional silver anisotropic nano-pinetree array modified indium tin oxide (Ag NPT/ITO)	Myo	10	10–5000	-	Spiked urine samples	[58]
Sandwich immunoassay conjugates of Ab-nanomaterials (AuNPs on the patterned paper microchannels; AuNPs labeled with MGITC); multiplex	CK-MB cTnI	7.92 × 10^−3^ 2.94 × 10^−3^	-	Interfering SERS signal in the presence of BSA, thrombin, and PSA	Spiked serum samples	[59]
LFA strips; sandwich immunocomplex; Au@AgAuNPs encoded with NBA	cTnI	0.09	-	CRP, BNP, Myo	-	[60]
LFA strips; sandwich immunoassay; Ab-conjugated Raman reporter embedded Ag@AuNPs; three test lines; multiplex	Myo cTnI CK-MB	3.2 × 10^−3^ 0.44 × 10^−3^ 0.55 × 10^−3^	1 × 10^−2^–5 × 10^2^ 1 × 10^−2^–50 2 × 10^−2^–90	-	Clinical human serum samples	[61]
LFA; Ab-conjugated Raman dyes encoded core–shell Ag-AuNPs; sandwich immunoassay; a single test line; multiplex	CK-MB cTnI Myo	0.93 × 10^−3^ 0.89 × 10^−3^ 4.2 × 10^−3^	2 × 10^−2^–90 2 × 10^−2^–90 1 × 10^−2^–5 × 10^2^	-	Clinical human serum from patients with AMI	[62]
Ab-conjugated nanomaterials (Au@Ag core–shell NPs labeled MGITC, gold-patterned chip); sandwich immunoassay; multiplex	cTnI CK-MB	8.9 × 10^−3^ 9.7 × 10^−3^	-	IgG, HSA, BSA, Myo and creatine kinase (CK)	Five clinical human serum samples from patients with AMI	[63]
Ab-conjugated nanomaterials (Raman encoded Ag-Au nanostars, Au-Ag-Au plasmonic array); sandwich immunocomplex; multiplex	cTnI NT-ProBNP NGAL IL-6 MMP-2 MMP-9	0.76 × 10^−6^ 0.53 × 10^−6^ 0.41 × 10^−6^ 1.3 × 10^−6^ 0.81 × 10^−6^ 0.75 × 10^−6^	1 × 10^−3^–1 × 10^3^	BSA, glucose, glutathione, IgG	Ten clinical human serum samples	[64]
LFIA; Ab-conjugated Raman reporter-embedded Au nanorod-core Au-shell nanotags	cTnT	0.1	-	-	-	[65]
paper-based immunoassay; gap-enhanced nanoparticles (GeNPs) based on Raman reporter-embedded into the gap between gold-core gold-shell NPs; multiplex	cTnI copeptin h-FABP	1 × 10^−2^ 4 × 10^−3^ 0.86	1 × 10^−2^–0.3 3 × 10^−2^–7.7 × 10^−2^ 4–52.3	some cross-reactivity among the three biomarkers	Spiked human serum	[66]
Aptamer-based sandwich assay on a paper strip; gold-core silica-shell nanoparticles	cTnI	1.6 × 10^−2^	1.6 × 10^−2^–1 × 10^−1^	CRP, BNP, h-FABP	Spiked human serum	[67]
Microfluidic paper-based device; Ab-conjugated Raman-encoded gold or silver nanoparticles enveloped in a silica shell; sandwich immunoassay, multiplex	GPBB cTnI CK-MB	8 × 10^−3^ 1 × 10^−3^ 1 × 10^−2^	Two linear dynamic ranges ≤ 1 ng/mL; ≥5 ng/mL	-	Clinical samples of human serum	[68]
Ab-conjugated magnetic beads; Ab-conjugated Raman- encoded hollow gold nanospheres; sandwich immunoassay; multiplex; magnetic separation	CK-MB cTnI	4.25 × 10^−2^ 3.37 × 10^−1^	-	-	Clinical human serum samples	[69]
CoFe_2_O_4_@AuNPs; SERS tags based on metal–organic frameworks @Au Tetrapods; sandwich immunosensor, magnetic purification	NT-proBNP	7.5 × 10^−7^	1 × 10^−6^–1 ng/mL	AFP, CEA, glucose, HSA, IgG	Spiked healthy human serum samples	[70]
Ab-conjugated materials (CoFe2O4@AuNPs, AFMOF-AuHPs-TB), microfluidic chip; sandwich immunosensor, magnetic separation	BNP	1 × 10^−3^	1 × 10^−3^–1 × 10^2^	-	-	[71]
Ab-conjugated (Raman reporter encoded AuNP–functionalized graphene oxide; magnetic beads); sandwich immunosensor	cTnI	5 × 10^−3^	1 × 10^−2^–1 × 10^3^	IgG, PSA, CEA, glucose	Spiked serum substitute media	[72]
Ab-conjugated magnetic beads; peptide-conjugated Raman reporter-encoded gold nanostars; sandwich immunocomplex; multiplexed detection; regeneration and reusability of the sensor; microfluidic device	cTnI NPY	5.5 × 10^−3^ 0.12	0.3–100	-	Spiked human serum samples from cardiac patients (11 samples)	[73]
Sandwich-based magnetic immunoassay; Ab-conjugated Raman embedded core–shell Au@Ag nanotags; streptavidin-magnetic beads; magnetic separation and concentration	cTnI	9.80 × 10^−3^	0–2	H-FABP, NT-proBNP, D-dimer, BSA	Fifty serum samples from AMI patients	[74]
Ab-conjugated Raman reporter embedded core–shell Au@Ag nanotags; streptavidin-magnetic beads; Sandwich-based magnetic immunoassay; multiplexed detection	cTnI H-FABP	4.4 × 10^−3^ 0.6396	0–1 0–100	NT-proBNP, D-dimer, BSA	Spiked diluted serum samples of healthy people	[75]
Aptamer-conjugated nanomaterials (Raman reporter encoded AuNPs, atomically flat Au nanoplates); sandwich-based immunocomplex	cTnI	2.4 × 10^−6^ (in buffer) 2.4 × 10^−3^ (in serum samples)	-	cTnC, cTnT, IgG, avidin	Nine clinical samples from both healthy humans and AMI patients	[76]
Ab-conjugated (polystyrene microspheres modified with AuNPs deposited on a silicon wafer; Raman reporter labeled AuNPs); sandwich immunocomplex; multiplex detection	cTnI CK-MB	3.16 × 10^−3^ 4.27 × 10^−3^	-	Myo, CEA, AFP, HSA, IgG	Spiked whole blood samples	[77]

NGAL: neutrophil gelatinase-associated lipocalin. LFA: lateral flow assay. LFTA: lateral flow immunoassay.

**Table 4:** Performances of recent fluorescence-based methods for the detection of CVD biomarkers.

Method Details	Excitation/Emission Wavelength (nm)	Analyte	LOD (ng/mL)	LR (ng/mL)	Selectivity Study	Analysis of Real Samples	Reference
LFIA; NaYF4: 30%Yb, 2%Er @NaLuF4 core–shell UCNPs	980/546	Myo	0.21	0.5–400 (DR)	CRP, BSA, NaCl, procalcitonin, hemolysis, high-bilirubin, high-cholesterol plasma	Clinical samples (plasma)	[80]
Aptamer-based homogeneous assay; fluorescein	495/517.6	Myo	0.020	0.050–100	CD63, BSA, EpCAM, and VEGF	Spiked human urine, saliva, serum	[82]
Pyrene-labeled aptamer; homogeneous assay	275/376	Myo	0.068	0.098–7.86	AFP, I, BSA, cTnI, IgA, and IgG	Spiked human sera	[87]
Quantum dot beads@SiO2-COOH (QBs@SiO2-COOH) nanobeads; lateral-flow immunoassay	365/620	CK-MB Myo cTnI	0.25 0.54 0.036	1.5–192 5–640 1–128	-	Human serum	[81]
LFIA, Time-resolved FRET; Donor: polystyrene raspberry nanoparticles coated with europium chelate modified silica nanoparticles; Acceptor: Au nanorods	340/615	cTnI	0.024 (PBS) 0.097 (Serum)	0.02–2 (PBS) 0.15–1.16 (serum)	-	Human serum	[83]
FRET carboxyfluorescein-modified aptamer; homogeneous assay	495/519	BNP	4.5 × 10^−5^	7.4 × 10^−5^–5.6 × 10^−4^	NT-proBNP, CRP, Myo, cTnI IFN, Cys, Gly, HSA, BSA, Arg, His	Blood	[85]
Homogeneous assay Fluorescein-tagged MIP	490	Myo	0.06	0.06–6 × 10^6^	-	Spiked fetal calf serum	[86]
FRET; aptamer-based homogeneous assay; CdSe/ZnS QD	375/655	CRP	0.045	0.05–1138	Transferrin, thrombin, TNF-alpha, albumin	Spiked and unspiked human serum	[88]
Cu-MOF with nanozyme activity and induced fluorescence upon reaction with H_2_O_2_; RNA-based homogeneous assay; dual fluorescence and colorimetry assay	320/410	CRP	0.24 (C) 0.04 (F)	0.5–50 (C) 0.1–50 (F)	glucose, glutathione, ascorbic acid, iron, creatinine, albumin, calcium	Spiked serum	[84]
Ab-based homogeneous assay; porous hydrogel with encapsulated photonic crystals (PhCs) barcodes; Cy-3 labeled antibodies	Not specified	cTnI BNP Myo	0.009 8.4 × 10^−5^ 0.68	1 × 10^−2^–1 × 10^3^ 1 × 10^−4^–10 1–1 × 10^4^	Mix of BNP and Myo (for cTnI) Mix of cTnI and Myo (for BNP) Mix of cTnI and BNP (for Myo)	Serum	[89]
Aptamer-based lateral flow assay; fluorescent microspheres	470/530	CK-MB	0.63	5–2 × 10^3^	cTnI, MB	Spiked serum	[90]
LFIA; Bodipy 650 labeled fluorescent latex microspheres; multiplex assay for 8 biomarkers from which CK-MB, cTnI and Myo by fluorescence; TC, TG, HDL-C, and UA by dry-chemistry; LDL-C is calculated	Not specified	CK-MB, cTnI Myo	2 0.001 0.01	Not specified	cTnI	Serum samples from AMI patients	[91]
MIP conjugated to CdTe QDs; imprinted hydroxyethylcellulose membrane	635/655	Myo	3.08 × 10^−3^	7.39 × 10^−3^–291 × 10^−3^	cTnT, creatinine, and HSA do not interfere at 10× higher concentrations than Myo	-	[92]
Homogeneous assay; dabcyl-modified aptamer and fluorescently (6-FAM) labeled cDNA;	495/517	Myo	0.07	0.1–5	BSA, AFP, IgA, IgG, HSA, and cTnI	Spiked human serum	[93]

LFIA: lateral flow immunoassay.UCNP: up-conversion nanoparticles. FRET: fluorescence resonance energy transfer. 6-FAM: 6-carboxyfluorescein). cDNA: complementary DNA. Dabcyl: (E)-4-((4-(dimethylamino) phenyl) diazenyl)benzoic acid. TC: cholesterol. TG: triglyceride. HDL-C: high-density lipoprotein cholesterol. UA: uric acid. LDL-C: low-density lipoprotein cholesterol. HSA: human serum albumin. AFP: alpha fetoprotein. Ig: immunoglobulin.

**Table 5:** Main features of chemiluminescence and electrochemiluminescence-based methods used in biosensors for the detection of CVD biomarkers.

Method Details	Assay Time	Analyte	LOD (ng/mL)	LR (ng/mL)	Selectivity Study	Analysis of Real Samples	Reference
Aptamer-MB/cTnI/Ab/anti-IgG Ab-HRP; microfluidic chip	30 min	cTnI	1.2 × 10^−2^	6 × 10^−2^–2.4 (buffer) 1.96 × 10^−1^–3.931 (serum)	BSA, NT-proBNP, and fibrinogen	Human serum	[94]
Ab1/biomarker/Ab2-HRP; microfluidic chip	17 min	cTnI, CK-MB, and Myo	cTnI: 1.02 × 10^−3^ CK-MB: 1.37 × 10^−3^, Myo: 4.15 × 10^−3^	cTnI: 2.0 × 10^−2^–2.560 CK-MB: 8 × 10^−2^–10.24 Myo: 0.8–2.048 × 10^2^	-	Human serum	[95]
ECL-RET; GCE/AgNC-sem@AuNPs-Ab/NT-pro-BNP/Ab-MIL125	>2 h	NT-proBNP	1.1 × 10^−4^	2.5 × 10^−4^–100	Β-amyloid PSA, PCT, CEA, insulin, AFP	Human serum	[96]

Ab1: capture antibody. Ab2: detection antibody. ECL-RET: electrochemiluminescence resonance energy transfer (RET). AgNC-sem@AuNPs: silver nanocubes coated with semicarbazide-modified gold nanoparticles. MIL-125: a Ti(IV)-based metal–organic framework. GCE: glassy carbon electrode. PSA: prostate-specific antigen, PCT: procalcitonin, CEA: carcinoembryonic antigen, AFP: αfetoprotein.

**Table 6:** Performances of some colorimetry-based approaches for the detection of CVD biomarkers.

Method Details	Assay Time	Analyte	LOD (ng/mL)	LR (ng/mL)	Selectivity Study	Analysis of Real Samples	Reference
AuNP/Apt;salt-induced aggregation of AuNPs	5 min	CRP	1.2 × 10^3^	8.89 × 10^2^–2.0 × 10^4^	BSA, aprotinin, proteinase K, L-glutamine, urea, ascorbic acid. BSA interferes at >100 nM	Spiked diluted human urine	[98]
Enzyme-free immunosorbent assay; Au nanovesicles with integrated allochroic dyes	>81 min	NT-proBNP CK-MB cTnT	7 × 10^−2^ 0.91 7.8 × 10^−3^	0.1–10^5^ 1–500 0.01–2	-	Human plasma pectoralgia patients and healthy individuals	[99]
MB/capture DNA/Apt/HRP-DNA1/DNA2	>165 min	Myo miRNA-141	8.75	0–7 × 10^3^	Gox, HSA, BSA, ALP	Spiked human serum	[97]
DNA hydrogel with encapsulated PtNPs/Cu-CPP(Fe); EXPAR combined with CRISPR-CAS14a	>100 min	CK-MB	0.355 pM	5 × 10^− 4^ nM–100 nM	cTnI, H-FABP, CRP, calcitonin	Spiked human serum samples	[100]
Microfluidic chip with DNA hydrogel with Apt/cDNA and embedded AuNPs	>3 h	CK-MB	0.147 (at 520 nm, C); 2.4 × 10^−3^ (coupling with microfluidic chip and cell phone as readout, M)	8.7–6.53·10^4^ (C) 17.4–4.875 × 10^4^ (M)	cTnI, Myocardial fatty acid binding protein (H-FABP), CRP, calcitonin	Spiked serum	[101]
Cu-MOF HRP-like nanozyme/Apt; dual colorimetry and fluorescence detection	>135 min (colorimetry-C) >10 h (fluorescence-F)	CRP	0.24 (C) 0.04 (F)	0.5–50 (C) 0.1–50 (F)	glucose, glutathione, ascorbic acid, iron, creatinine, albumin, calcium	Spiked serum	[84]
Microfluidic chip; AB/Ab1/biomarker/Ab2-biotin/streptavidin-HRP	20 min	cTnI hFABP NT-proBNP	9.56 × 10^−3^ 95.5 × 10^−3^ 5.29 × 10^−3^	QL: 28 × 10^−3^ 0.290 16.04 × 10^−3^	-	Spiked plasma; samples from healthy + patients with ACS, DCM and AS	[102]
Paper microfluidic device; sandwich immunoassay; conjugates of Ab-nanomaterials (AuNPs, AgNPs, Au urchin)	10 min	GPBB CK-MB cTn T	0.5 0.5 0.05	0–100 0–100 0–200	HSA, uric acid, ascorbic acid	Clinical human sera	[103]
LFIA; Ab-AuNPs conjugates	1.5 min	MB CRP DDm	30 300 300	30–3 × 10^3^ 3 × 10^2^–3 × 10^4^ 3 × 10^2^–1 × 10^5^	No cross reactivity with the other biomarkers	Human serum	[104]
LFIA, Ab, HRP mimicking nanozyme (Au@Ag-Pt NPs) conjugate	10 min	CRP	1.5 × 10^−2^ in serum	-	Serum albumin, IgG, procalcitonin, cTnI cTnT	Spiked rabbit serum	[105]
Sandwich assay citicoline- BSA/CRP/Apt-AuNPs (AuNPs as HRP mimicking nanozyme)	≈80 min	CRP	8 × 10^−6^	0.1–200	Myo, cTnI, growth differentiation factor 15, BSA, γ-globulin, non-fat milk powder, aspartic acid, arginine, glycine, glucose, fibrinogen, transferrin.	Rat serum; spiked rat serum	[106]
ELONA (direct and sandwich); SA/biotinin-Apt/streptavidin-HRP	2.5–3 h	cTnT	3.42 nM (direct) 3.13 nM (sandwich)	-	Non-specific adsorption observed for undiluted serum (direct assay)	Human serum	[107]
Microfluidic paper; Ab1/cTnI/Ab2/H1/hemin (DNAzyme)	45 min	cTnI	1 × 10^−3^	5 × 10^−3^–1 × 10^2^	HSA, Hb, CEA, AFP	Spiked serum	[108]
Glass plate/MOF-818 nanozyme confined in porous WO_3_/Apt-Glu/catechol oxidase-mimic, Exo-I assisted signal amplification	>30 min	cTnI	1.8 × 10^−5^	5 × 10^−5^–100	CRP, Myo, HSA, IgG, CEA, AFP.	Spiked serum, unspiked serum	[109]

HRP: horseradish peroxides; Apt: aptamer.MB: magnetic beads; Myo: Myoglobin; Gox: glucose oxidase (Gox); HSA, human serum albumin; BSA: bovine serum albumin. ALP: alkaline phosphatase. AB: antibody. hFABP: heart-type fatty acid binding protein. ACS: acute coronary syndrome. DCM: dilated cardiomyopathy. AS: aortic stenosis. QL: limit of quantitation. GPBB: glycogen phosphorylase isoenzyme. LFIA: lateral flow immunoassay, Hb: hemoglobin, CEA, carcinoembryonic antigen. AFP: α-1-fetoprotein. ELONA: enzyme-linked oligonucleotide assay. Cu-TCPP(Fe): metallic organic framework with Fe (III) meso-tetra(4-carboxyphenyl) porphine chloride (TCPP-(Fe) and Cu. PtNPs; Pt nanoparticles. EXPAR: exponential amplification reaction. CRISPR: Clustered regularly interspaced short palindromic repeat

**Table 7:** Examples of other optical detection methods used in biosensors for the detection of CVD biomarkers.

Method Details	Assay Time	ANALYTE	LOD (ng/mL)	LR (ng/mL)	Selectivity Study	Analysis of Real Samples	Reference
Retroreflection; Ab-coated Si-based Janus particles; sandwich-type assay	>40 min	CK-MB	4 × 10^−1^	4 × 10^−1^–1 × 10^3^	CK-MM	Spiked human serum	[111]
Dynamic light scattering; aggregates formed by Ab-MB and SiO_2_@PBA-aminophenylboronic acid; sandwich-type assay	20 min	NT-proBNP	7.4 × 10^−6^	1.2 × 10^−5^–1 × 10^−1^	CEA, AFP, HCG, HBsAg, Glu, Gal, Fuc, NeuAc	Clinical samples	[112]
Microfiber Bragg grating; Ab-coated fiber; direct detection	25 min at 37 °C	cTnI	13.5	13.5–1 × 10^3^	CEACAM; AFP	Human serum	[113]

CK-MM: an isoenzyme of creatine kinase-myocardial band; AB-MB: antibody coated magnetic particles. SiO2@PBA: silica particles modified with 3-aminophenylboronic acid. CEACAM 5: carcinoembryonic antigen-related cell adhesion molecule 5; AFP: alphafetoprotein. CEA: carcinoembryonic antigen. HCG: human chorionic gonadotropin HBVAg: hepatitis B virus antigen. Glu: glucose. Gal: galactose. Fuc: fucose. NeuAc: n-acetylneuraminic acid.

**Table 8:** Main features of optical detection methods used in biosensors for the detection of CVD biomarkers.

Method	Analyte	LOD (ng/mL)	Reference	Advantages	Disadvantages
SPR	cTnT	5.25 × 10^−5^ (15 aM)	[44]	Enables the monitoring of ligand binding in real time; Label-free	Further sensitivity enhancement requires amplification systems that complicate the measurement
SERS	cTnI	7.6 × 10^−7^	[62]	Sensitive, down to single molecule, non-destructive; multiplexing enabled by using various SERS reporters	Chemometrics needed for interpreting and denoising complex spectra; reproducibility depending on the substrate preparation
Fluorescence	cTnI	1 × 10^−3^	[91]	Multiplexing enabled by a high variety of fluorophores; implemented in clinical practice	Sensitive to interferences due to the background fluorescence of proteins present in high concentration in biological samples
Colorimetry	cTnI	1.8 × 10^−5^	[109]	Simple, fast, low costs, simple or no instruments, compatible with LFIA/smartphone	Not very sensitive; sensitivity enhancement presumes more-complicated or costlier analysis
Retroreflection	CK-MB	4 × 10^−1^	[111]	Simplified optical equipment, use of polychromatic light	Retroreflective particles for sensors not commercially available
Light scattering	NT-proBNP	7.4 × 10^−6^	[112]	Very low (<3 µL) amounts of sample needed; fast (<2 min)	Highly influenced by temperature and viscosity
Chemiluminescence	cTnI	1.02 × 10^−3^	[95]	Sensitive, fast; wide detection range; compatible with automated equipment and implemented in clinical laboratories	Requires the addition of reagents to induce the emission of luminescence; costs can be important
Microfiber Bragg grating	cTnI	13.5	[113]	Potential for in vivo and at-patient monitoring of biomarkers; compactness; multiplexing possibilities	Need for surface regeneration; the costs are significant; temperature needs to be controlled

## Data Availability

Not applicable.
